# Undetected antisense tRNAs in mitochondrial genomes?

**DOI:** 10.1186/1745-6150-5-39

**Published:** 2010-06-16

**Authors:** Hervé Seligmann

**Affiliations:** 1 Center for Ecological and Evolutionary Synthesis, Department of Biology, University of Oslo, Blindern, 3016 Oslo, Norway; 2 Department of Evolution, Systematics and Ecology, The Hebrew University of Jerusalem, 91904, Israel

## Abstract

**Background:**

The hypothesis that both mitochondrial (mt) complementary DNA strands of tRNA genes code for tRNAs (sense-antisense coding) is explored. This could explain why mt tRNA mutations are 6.5 times more frequently pathogenic than in other mt sequences. Antisense tRNA expression is plausible because tRNA punctuation signals mt sense RNA maturation: both sense and antisense tRNAs form secondary structures potentially signalling processing. Sense RNA maturation processes by default 11 antisense tRNAs neighbouring sense genes. If antisense tRNAs are expressed, processed antisense tRNAs should have adapted more for translational activity than unprocessed ones. Four tRNA properties are examined: antisense tRNA 5' and 3' end processing by sense RNA maturation and its accuracy, cloverleaf stability and misacylation potential.

**Results:**

Processed antisense tRNAs align better with standard tRNA sequences with the same cognate than unprocessed antisense tRNAs, suggesting less misacylations. Misacylation increases with cloverleaf fragility and processing inaccuracy. Cloverleaf fragility, misacylation and processing accuracy of antisense tRNAs decrease with genome-wide usage of their predicted cognate amino acid.

**Conclusions:**

These properties correlate as if they adaptively coevolved for translational activity by some antisense tRNAs, and to avoid such activity by other antisense tRNAs. Analyses also suggest previously unsuspected particularities of aminoacylation specificity in mt tRNAs: combinations of competition between tRNAs on tRNA synthetases with competition between tRNA synthetases on tRNAs determine specificities of tRNA amino acylations. The latter analyses show that alignment methods used to detect tRNA cognates yield relatively robust results, even when they apparently fail to detect the tRNA's cognate amino acid and indicate high misacylation potential.

**Reviewers:**

This article was reviewed by Dr Juergen Brosius, Dr Anthony M Poole and Dr Andrei S Rodin (nominated by Dr Rob Knight).

## Background

The genetic code maximizes both the number of off-frame stop codons and the ability to form secondary structure by coding sequences [1]. This suggests that coding sequences have multiple functional dimensions, beyond that of linear coding. Indeed, life history and other fitness-related properties associate with each of these non-coding, yet non-random properties of mRNAs: off frame stop frequency [2,3]; and secondary structure formation [4-7]. These observations suggest that sequences should be considered from a perspective of fulfilling multiple tasks. The fact that specific sequences have multiple functions increases the difficulty of estimating the number of functional sequences [8-11].

Considering the possibility of multiple functions could explain some discrepancies from the neutral theory of evolution. Analyses presented here suggest an additional function for mitochondrial tDNA, that of antisense coding [12]. Such complementary strand (sense-antisense) coding is likely for some proteins [13,14], at least in the ancestral coding system [15-17]. Some evidence also suggests it occurred for tRNAs, where tRNAs with complementary anticodons were presumably coded by complementary strands [18-20]. This is suggested, among others, by complementarities existing between acceptor stems of these tRNA pairs [21].

In human mitochondrial tRNAs, about 35% of known polymorphisms are associated with diseases, as compared to 6% for sequences in the mitochondrial genome that do not code for tRNAs (data in appendix of [22], from [23-25]). This puzzle might be due to the fact that mitochondrial tRNA genes have at least two functions in addition to coding for tRNAs: they apparently function as replication origins [26-29], and hybridization between the expressed tRNA and its complementary tDNA regulates replication origin formation by the tDNA [22]. The property examined here, antisense coding, could be an additional fourth function for mitochondrial tRNA genes.

Antisense RNAs usually function as repressors of the sense RNA by hybridizing with their sense counterpart, preventing its usual activity as single-stranded RNA [30,31]. However, for tRNAs, the antisense molecule could have a classical tRNA function in translation, because antisense tRNAs frequently form cloverleaf secondary structures [32,33]. This hypothesis is particularly relevant for vertebrate mitochondrial genomes: both strands are transcribed in their entirety and matured according to recognition of secondary structures formed by tRNA sequences in the long unprocessed RNA transcript, a process called RNA maturation by tRNA punctuation [34,35]. In addition, sense-antisense coding is also more likely in organisms with reduced genomes [36], such as mitochondria.

Because sense RNA processing depends on secondary structure formation by sense tRNA sequences, the same process probably works for antisense tRNAs. This is an obvious outcome of the complementary nature of DNA or RNA double strands. However, processing by tRNA punctuation is probably less frequent for antisense tRNAs than for sense tRNAs because cloverleaf stabilities of antisense tRNAs (estimated by the structural component of COVE calculated by tRNAscan-SE [37], http://lowelab.ucsc.edu/tRNAscan-SE/, an online software designed to detect tRNAs) are generally weaker than stabilities of their sense counterparts. Note that COVE is not estimating thermodynamic stability, but is an information-based index that measures covariation between parts of the sequence, in relation to a generalized tRNA covariance model [38]. COVE estimates the potential for cloverleaf formation. It is used here as an estimate of potential capacity for cloverleaf formation by the presumed tRNA sequence. Positive COVE values indicate that the sequence has a greater tendency to form a cloverleaf structure than random sequences, and negative values less tendency than random sequences.

**Figure 1:**
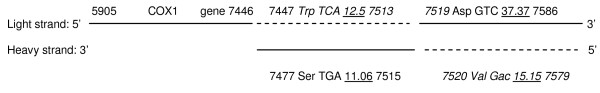
**Scheme of the human mitochondrial genome region templating for sense tRNAs Ser UGA and Asp GUC**. Continuous lines indicate expressed sense genes, dashed lines hypothetically expressed antisense genes. tRNAs are indicated by their cognate amino acid, followed by the anticodon detected by tRNAscan-SE http://lowelab.ucsc.edu/tRNAscan-SE/ and the structural component of the Cove index of that tRNA. Hypothetical antisense tRNAs are in italics. Gene positions on the mitochondrial genome are indicated according to the standard in Genbank (NC_012920). The 5' flank of the antisense of tRNA Ser UGA is processed by sense RNA maturation by default because it is flanked by the mitochondrial protein coding gene COX1, its 3' flank because of its vicinity with sense tRNA Asp GUC. Normal sense RNA maturation processes only the 3' flank of the hypothetical antisense of sense tRNA Asp GUC. Extremities of tRNAs and their anticodons are as detected for these sequences by tRNAscan-SE.

Regular mitochondrial sense RNA maturation processes both 5' and 3' extremities of two antisense tRNAs (tRNA Gln and tRNA Ser UCN), and of 5 and 4 antisense tRNAs at their 5' and 3' extremities, respectively (5': Glu, Ile, Thr, Trp, Tyr; 3': Ala, Asp, His, Pro). This is schematically explained in Figure 1. This scenario assumes that antisense tRNAs are not processed because of their own capacity to form secondary structures punctuating RNA processing, but because of tRNA punctuation of sense RNA maturation by sense tRNAs. Independently of whether processing of antisense tRNAs occurs because their own secondary structure signals processing, or because of signalling by sense tRNAs, some experimental evidence confirms their existence: mitochondrial antisense tRNAs have sometimes been detected [39]. This includes antisense tRNAs that are not processed by normal sense RNA maturation and would depend on their own capacity to form secondary structures for punctuation of RNA processing [40-43]. Functioning as tRNAs in translation requires that these tRNAs are loaded with an amino acid. It seems reasonable that this occurs, because even RNA corresponding to the mitochondrial light strand replication origin, OL, which is apparently occasionally transcribed into RNA and forms a stem-loop hairpin secondary structure, is aminoacylated [44]. Analyses of anticodons expected for antisense tRNAs also suggests translational activity by antisense tRNAs: genomes avoid sense tRNAs whose anticodons define by complementarity antisense anticodons that match stop (terminator) codons, and hence would form, if transcribed and processed, antiterminator (termination suppressor) tRNAs [45]. Antitermination activity would profoundly impair protein synthesis. Hence the observation that putative antisense antiterminator tRNAs are avoided suggests that antisense tRNAs have at least occasional translational activity. Indeed, in about 85% of the vertebrate mitochondrial tRNAs examined, the anticodon of the antisense tRNA (as detected by tRNAscan-SE) is defined by the exact inverse complement of the sense anticodon [46]. The major exceptions to this rule of anticodon symmetry are for the antisense anticodons of sense tRNAs Leu CUN and UUR which would match stop codons UAG and UAA, respectively. For these tRNAs, the antisense sequences form cloverleaf structures with anticodons that are usually not the inverse complements of the sense anticodons. Furthermore, when antisense antiterminator anticodons are not avoided, translational activity by the antisense tRNA is minimized by the fact that antisense antiterminator tRNAs form usually weaker cloverleaf structures than homologous antisense tRNAs with different anticodons. Also, genomes possessing a given antisense antiterminator tRNA tend to avoid using stop codons matching that antiterminator anticodon [46].

### Normal processing of antisense tRNAs

It seems probable that because antisense tRNAs form weaker cloverleaves, antisense tRNAs depending only on their own structural signal for processing are on average less processed. Hence comparing the group of 11 antisense tRNAs processed by regular sense RNA maturation to the group of antisense tRNAs depending only on their own processing signal is a natural system on which to test whether the first group, as compared to the second group, is better adapted for translational activity. Here I show evidence that various properties of antisense tRNAs that are important for translation correlate with one another in a way compatible with antisense tRNA translational activity.

One can expect that mitochondrial antisense tRNAs processed during regular sense RNA maturation (processed because of the structural signal by sense tRNAs, not their own) are more expressed than those usually unprocessed by sense RNA maturation. Therefore, antisense tRNAs processed by sense RNA maturation should more frequently function during translation, and their properties in relation to translation should resemble more sense tRNAs than the same properties of usually unprocessed antisense tRNAs. These processed antisense tRNAs hence probably routinely expand the known mitochondrial sense tRNA pool and should be adapted for translation. This will be termed here the extension hypothesis. Usually unprocessed antisense tRNAs are probably more rarely processed, and would presumably only occasionally function in translation. They are expected to be, on average, less similar to regular tRNAs, hence translational activity by these antisense tRNAs should be avoided. This will be termed here as the (antisense tRNA) avoidance hypothesis. Both hypotheses are adaptive and independent, and probably coexist, each for different tRNA species (not necessarily in full association with sense RNA maturation), suggesting a gradient between two extremes, antisense tRNAs that probably frequently extend the tRNA pool, and those whose translational activity should be avoided. I analyze here properties relevant to translation of the antisense sequence of 49 primate taxa for which complete mitochondrial genomes are available in GenBank (October 2009). Analyzes, among others, test whether processed versus unprocessed antisense tRNAs differ in these properties. The antisense tRNA properties explored are antisense tRNA misacylation, cloverleaf fragility, and the inaccuracy of antisense processing by sense RNA maturation (for the 11 antisense tRNAs where this occurs). These properties are expected higher in unprocessed than processed antisense tRNAs. Each property is described separately, and pairwise associations between all six combinations of these four properties are explored. Correlations of these antisense tRNA properties with the genome-wide usage of their cognate amino acid are also explored. Results from these analyses, combined in a meta-analysis, suggest that some antisense tRNAs extend the tRNA pool.

## Results

### Antisense anticodon prediction from cloverleaf secondary structure

In order to estimate the extent by which tRNAs adapted to function in translation, predictions of the amino acids that match their anticodons according to the mitochondrial genetic code are required. This amino acid is usually termed 'cognate', the 19 others are non-cognates. The secondary structure of a tRNA, predicted by tRNAscan-SE, predicts the most probable anticodon for tRNA sequences. Using the vertebrate mitochondrial genetic code, that anticodon predicts the cognate of that antisense tRNA. Table 1 describes cognate amino acids, anticodons and secondary structure stabilities (estimated by the structural component of COVE output by tRNAscan-SE) of the 22 most common (modal) human mitochondrial sense and antisense tRNAs (sequences are from the appendix of [22]). Similar analyses were done for the mitochondrial genomes of 48 other primates for which complete mitochondrial sequence data were available at GenBank (October 2009). tRNAscan-SE detects correctly all 21 sense anticodons of human mitochondrial sense tRNAs that form classical cloverleaves (the 22^nd^, tRNA Ser AGY, is excluded from this analysis as it does not form a classical cloverleaf (it lacks the tRNA's D-arm) and is undetectable by that software). The software was set to its mitochondrial/chloroplast (organelle) mode. It used for anticodon definition the vertebrate mitochondrial genetic code. For each species, the sense tRNA sequences extracted by tRNAscan-SE were inverse complemented and processed by tRNAscan-SE, defining its COVE cut-off score for tRNA detection to COVE = -100. For tRNA Ser AGY, the sequences used were as those annotated in GenBank for that genome. For *Homo sapiens*, tRNAscan-SE did not detect antisense anticodons in 5 antisense tRNAs (23%). In these 5 tRNAs, the anticodon remains ambiguous for the antisense tRNA. In 15 among the 16 remaining antisense tRNAs (94%), the antisense anticodon was precisely the antisense sequence of the sense anticodon (e.g., for sense anticodon UCA, which codes for Trp, the antisense anticodon is UGA, which codes for Ser). This was also the case for 70% of 50 different *E. coli *tRNAs (NC010468). Hence the sense tRNA's anticodon usually predicts the antisense tRNA's anticodon. This property is termed anticodon symmetry between sense and antisense tRNAs. The only human antisense tRNA lacking anticodon symmetry was the antisense of tRNA Leu CUN. In this case, anticodon symmetry would have resulted in an antitermination anticodon which matches a stop codon. The fact that precisely in this case there is no anticodon symmetry could also be an adaptation to avoid detrimental effects of antisense tRNA expression. This property of avoiding antitermination antisense tRNAs by anticodon asymmetry is illustrated and explored in more detail elsewhere [46].

**Table 1:** Anticodons of sense and antisense tRNAs in vertebrate mitochondria for heavy- and light-strand encoded sense tRNAs and cognate amino acid matching sense and antisense anticodons according to the vertebrate mitochondrial genetic code.

Sense tRNA			Antisense tRNA				
**Cognate**	**Anticodon**	**COVE**	**Anticodon**	**Cognate**	**COVE**	**Processing**	**Dist**.

Heavy strand							
							
Ala	TGC	23.03	GCA, undet	Cys, ?	1.9	3'	8
Asn	GTT	18.2	AAC, undet	Val, ?	12.04	none	
Cys	GCA	20.27	TGC	Ala	-7.83	none	
Gln	TTG	18.53	CAA	Leu UUR	-20.71	5', 3'	4
Glu	TTC	22.46	GAA	Phe	3.56	5'	5
							
Pro	TGG	30.29	CCA	Trp	16.31	3'	2
Ser UCN	TGA	11.06	TCA	Trp	12.5	5', 3'	2
Tyr	GTA	16.92	TAC	Val	-1.55	5'	13
Light strand							
							
Arg	TCG	28.22	CGA	Ser UCN	21.2	none	
Asp	GTC	37.53	GAC	Val	15.15	3'	2
Gly	TCC	37.42	GGA	Ser UCN	18.42	none	
His	GTG	21.64	CAC	Val	16.99	none	
Ile	GAT	29.27	ATC	Asp	10.84	5'	2
Leu UUR	TAA	21.47	TTA, undet	Stop, ?	-15.65	none	
Leu CUN	TAG	30.67	CTA, TAA	Stop, Leu UUR	3.56	none	
							
Lys	TTT	33.39	AAA, undet	Phe, ?	21.53	none	
							
Met	CAT	12.41	ATG	His	11.71	3'	2
Phe	GAA	17.72	TTC	Glu	4.61	none	
Ser AGY	GCT		AGC, undet	Ala, ?	4.75	none	
							
Thr	TGT	28.23	ACA	Cys	9.87	5'	2
Trp	TCA	31.2	TGA	Ser UCN	3.35	5'	8
Val	TAC	21.88	GTA	Tyr	6.78	none	

The 3d and 6^th ^columns indicate COVE scores for the secondary structure component of the tRNA's cloverleaf, calculated by tRNAscan-SE (http://lowelab.ucsc.edu/tRNAscan-SE/[37]). The 4th column indicates the antisense anticodon as the inverse complement of the sense anticodon in column 2. If tRNAscan-SE detected a different (or no) anticodon, this is indicated after the comma. The 5th column indicates the amino acid matching the antisense anticodon according to the vertebrate mitochondrial genetic code. Column 7 indicates whether normal mitochondrial RNA maturation processes the 5' and/or 3' extremities of the antisense tRNA. The last column indicates the distance in number of nucleotides between these extremities of the antisense tRNA and the sense gene processed by regular sense RNA maturation

### Antisense tRNAs resemble regular tRNAs with the same cognate amino acids

Proper tRNA translational activity requires tRNA aminoacylation by the tRNA's cognate, which is the amino acid that matches the tRNA's anticodon according to the genetic code. Aminoacylation (also termed tRNA loading) is done by class I and class II aminoacyl tRNA synthetases, two protein groups specific to different cognate amino acids that aminoacylate tRNA acceptor stems with their cognate. Note that symmetries in the genetic code determine which amino acid is amynoacylated by which tRNA synthetase class [47,48], a fact that might be relevant to sense-antisense tRNA pairs [21].

These tRNA synthetases recognize tRNAs according to yet undefined nucleotide patterns on the tRNA, mainly in the tRNA's acceptor stem [49]. Misloading of antisense tRNAs would result from tRNA nucleotide patterns that confer information that is not matched with the tRNA's anticodon, hence a lack of coadaptation between the anticodon and the rest of the tRNA. If such coadaptation between parts of the (antisense) tRNA exists, it is evidence that the antisense tRNA is adapted for translation, confirming the extension hypothesis. The 'code' according to which anticodons (and cognates) are matched with other parts of the tRNA is not yet well elucidated [50], but some association apparently exists between anticodon and acceptor stem nucleotide contents [51].

A sequence alignment approach is usually used to detect nucleotides (other than anticodons) determining correct aminoacylation [52]. The online available bioinformatic application 'TFAM' [53]http://tfam.lcb.uu.se/ aligns target (focal) tRNA sequences to large datasets of known functional groups of tRNA sequences, mainly of bacterial origin. TFAM uses the latter sequences as reference sequences, and outputs scores that estimate the quality of the alignment of the input, focal sequence with each of the known functional reference tRNA groups. A positive alignment score with one of the reference tRNA groups with known cognate indicates that the alignment of the focal sequence with that group of tRNAs is better than for random sequences. Negative scores indicate that the alignment is worse than for random sequences. Supposedly, a tRNA sequence has greater aminoacylation potential with the cognate of the TFAM-tRNA functional group that aligns best with it. Because this procedure depends on the complete tRNA sequence, it includes, and corresponds mainly to sequence information besides the anticodon. Scores in the TFAM output are therefore interpreted here as proportional to the aminoacylation potential of specific amino acids for that target tRNA sequence. According to the context, these scores are used here according to the interpretation that they reflect aminoacylation potential, or as a direct measure of alignment quality with the 'standard' bacterial tRNAs used as reference sequences by TFAM.

Table 2 presents the TFAM-scores obtained for sense and antisense tRNAs versus each functional tRNA group for the 22 human mitochondrial tRNA sequences from [22]. I quantify the potential for misacylation of a tRNA by the number of non-cognates with TFAM scores higher than the tRNA's cognate amino acid predicted by tRNAscan-SE according to the structure of the anticodon loop. For antisense tRNAs for which no anticodon was detected, I use the principle of anticodon symmetry between sense and antisense tRNAs: the inverse complement of the sense anticodon is used to predict the antisense tRNA's cognate amino acid. Only results for *Homo sapiens *are presented in detail here, but all 49 primate genomes included in this study were analysed as described here by tRNAscan-SE and TFAM.

**Table 2:** TFAM scores for alignments of human sense and antisense mt tRNA sequences with groups of tRNAs with known function.

*tRNA*	*A*	*C*	*D*	*E*	*F*	*G*	*H*	*I*	*K*	*L*	*M*	*N*	*P*	*Q*	*R*	*S*	*T*	*V*	*W*	*Y*
Sense																				
Ala, 11	**-19**	-17	-19	-22	-30	1	-10	-57	0	-48	-21	-7	-4	5	-5	-79	-3	-3	3	-34
Cys, 1**	-42	**15**	8	5	-5	-1	-2	-34	-5	-33	-13	8	0	4	10	-69	-8	-14	18	-26
Asp, 9	-39	-3	**-13**	-8	-33	9	-12	-29	-7	-66	-29	-19	-12	-15	-8	-70	-15	-2	-2	-44
Glu, 6	-34	-19	-17	**-10**	-7	-16	-16	-38	-7	-46	-30	-8	-32	0	-8	-82	-11	-11	1	-42
Phe, 2**	-35	-12	-15	-15	**7**	-7	-9	-15	-7	-48	15	-12	-12	-22	5	-56	10	-6	-14	-26
Gly, 5**	-35	1	-14	8	-8	**4**	-4	-42	9	-46	-31	3	-27	9	-2	-75	7	-1	7	-38
His, 1**	-27	-13	-16	-3	-20	15	**13**	-53	-17	-55	-22	-14	-26	-6	-3	-57	-12	-17	-13	-28
Ile, 14	-28	-9	-17	-19	-16	3	-6	**-23**	-24	-46	-3	-22	-9	-21	-13	-46	-13	-1	-7	-35
Lys, 0**	-10	1	-4	-8	-1	17	0	-29	**19**	-49	0	2	1	-10	11	-70	9	9	2	-31
LeuUUR, 17**	-49	-18	-38	-21	-10	-29	-20	-43	-23	**-48**	-11	-30	-25	-32	-17	-61	-21	-19	-32	-38
Leu CUN, 16**	-46	6	-19	-7	-4	-8	-2	-32	-10	**-34**	-21	-12	-17	14	-3	-72	-11	-9	1	-45
Met, 0**	-37	-6	-16	-19	-11	-22	-13	-36	-10	-55	**1**	-8	-21	-3	-11	-66	-23	-11	1	-32
Asn, 0**	-34	-23	-4	-28	-35	-21	-2	-25	-11	-77	-21	**4**	-10	-8	0	-80	-23	-13	-14	-55
Pro, 1**	-23	-37	-24	-30	-25	15	-8	-31	-9	-45	-26	-25	**8**	-10	-1	-86	-16	2	4	-61
Gln, 0**	-42	-22	-27	-17	-29	-17	-1	-69	-26	-33	-30	-15	-14	**2**	-17	-79	-15	-35	-4	-52
Arg, 5**	-25	-6	-15	-12	-13	3	-7	-33	-3	-53	-16	-3	-8	28	**-5**	-60	-9	-9	9	-31
SerAGY, 19**	1	38	18	21	21	37	37	12	45	-19	30	36	30	36	28	**-31**	30	35	34	9
SerUCN, 17**	-28	-11	-16	-13	-22	-20	-15	-53	-8	-51	-17	-4	-15	-8	-6	**-40**	-16	-11	-2	-7
Thr, 2**	-27	-8	-4	4	-6	6	-8	-25	0	-38	-15	-2	-18	-4	4	-65	**5**	-13	11	-36
Trp, 0**	-12	6	-6	3	-4	16	-7	-34	6	-49	-4	0	-8	6	8	-38	-2	1	**27**	-32
Tyr, 16**	-24	-6	-19	-25	-5	-10	-12	-33	-9	-46	-7	-10	-19	-25	-10	-51	-4	-7	-15	**-28**
Val, 2**	-18	-10	-17	-21	-16	4	-13	-30	-14	-47	-18	-13	-6	-8	-7	-82	-5	**-4**	-2	-44
																				
Antisense	A	C	D	E	F	G	H	I	K	L	M	N	P	Q	R	S	T	V	W	Y
Cys, 0**	23	**53**	26	41	29	35	46	-11	38	-27	20	33	36	42	29	-41	24	40	46	13
Ala, 16**	**-11**	34	17	32	27	15	29	-28	28	-18	3	18	3	20	18	-27	14	11	15	3
Val, 2**	-13	14	10	7	-1	40	10	-26	-1	-55	-5	3	2	6	-1	-40	5	**18**	19	-21
Phe, 1**	-14	38	8	5	**36**	-2	21	1	23	-31	12	28	4	20	21	-39	20	18	33	-8
Glu, 5**	-2	31	35	**35**	24	27	58	-22	42	46	2	30	24	54	16	5	19	31	41	31
SerUCN, 19**	-25	16	4	8	12	-5	8	-31	15	-34	-11	23	-11	6	-1	**-61**	16	2	3	-18
Val, 2**	0	27	18	21	21	23	35	-2	29	-24	20	15	20	32	21	-22	10	**31**	31	16
Asp, 7	-8	40	**23**	31	13	27	38	-16	23	-28	11	22	21	33	14	-9	7	39	29	17
Phe, 8	-33	20	21	-2	**10**	1	14	-35	11	-2	-16	16	-6	20	-3	-44	2	11	26	-4
Stop, 20**	-19	40	26	12	26	5	30	-30	17	-1	5	36	12	25	7	-13	11	11	29	23
LeuUUR, 17**	-27	10	-1	14	6	-10	-1	-38	19	**-29**	-12	21	6	15	6	-61	3	4	7	-19
His, 4**	-13	18	12	5	10	13	**27**	-8	13	-13	-5	9	28	24	7	-36	7	16	15	-12
Val, 6	-15	30	18	14	8	27	34	-22	7	-29	3	27	24	38	8	-47	9	**19**	19	-11
Trp, 4**	-4	47	23	41	23	23	39	-15	33	-21	19	21	27	39	28	-27	14	25	**38**	23
LeuUUR, 15**	-22	25	-14	-4	15	6	16	-42	11	**-9**	3	8	-1	11	-4	-26	4	3	20	-5
SerUCN, 20**	0	36	13	5	36	19	32	-17	25	-26	13	30	18	35	15	**-34**	32	21	37	12
Ala, 12	**24**	39	32	18	36	28	48	12	25	-14	22	34	49	34	21	-35	31	41	37	0
Trp, 4**	-16	20	2	5	2	-5	17	-47	19	-20	-14	11	10	25	-5	-29	-5	7	**12**	6
Cys, 0**	-14	**29**	17	22	1	-7	14	-15	11	-41	-13	14	2	26	16	-37	1	10	24	-3
Tyr, 12	-13	4	8	-7	9	6	19	-31	10	-36	-6	21	-12	12	-2	-50	8	9	2	**-5**
SerUCN, 19**	-12	16	-6	3	0	17	9	-41	1	-32	-17	3	1	17	-7	**-43**	9	10	1	-7
Val, 3**	-16	22	12	14	2	-5	18	-17	18	-33	-5	14	15	35	0	-18	-6	**18**	22	9

Each row is for a target tRNA sequence, indicated by its three letter cognate abbreviation, followed by the number of scores higher than the score of the cognate predicted by the anticodon. Columns are headed by the standard one letter abbreviation of cognate amino acids, indicating the known tRNA functional groups. Scores are alignment quality (here interpreted as proportional to probability of acylation of the target tRNA sequence by the cognate of the 'standard' tRNA group used by TFAM (http://tfam.lcb.uu.se/[53])) between the target tRNA sequence and the sequences used by TFAM as the reference group of tRNAs with known function. Negative and positive scores indicate alignment quality lower and higher than for random sequences, respectively. Bold scores indicate the amino acid matching the target tRNA's anticodon according to the genetic code. Antisense tRNAs are ordered according to their matching sense tRNA. Asterisks indicate two tailed statistical significance at P < 0.05 according to a binomial distribution for misacylation potentials.

It is known that alignment procedures frequently do not succeed in determining cognates of sense mitochondrial tRNAs. It seems that the mitochondrial tRNA acylation 'code' is not well elucidated. This is reflected by the fact that TFAM detects the same amino acid as predicted by the anticodon in only 5 sense and 2 antisense human mt tRNAs (sense, 23%: Asn, Gln, His, Lys and Met; antisense, 10%: Ala, Thr). This is not the case for *E. coli*: TFAM detects correctly the cognate in 96% of the 50 sense tRNAs, but only 2% of its antisense tRNAs.

**Figure 2:**
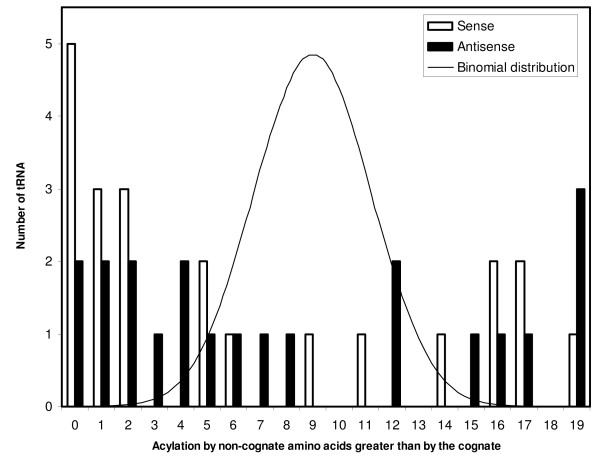
**Frequency distribution of misacylation tendency for sense and antisense tRNAs (white and black columns, respectively)**. The modal (most frequent) human tRNA sequence was used for each of the 22 mitochondrial tRNA species. TFAM aligns each sequence to reference tRNA sequences with known cognates. Alignment quality with each tRNA reference estimates the tendency of the focal tRNA sequence for acylation with the cognate of the tRNA reference (raw data in Table 2). The x axis indicates the number of tRNA references with cognates differing from the focal tRNA's cognate but aligning better with the focal tRNA sequence than the reference tRNA with the cognate matching the focal tRNA's anticodon. Hence numbers close to zero on the x axis indicate low tendency for misacylation. The y axis is the number of focal tRNAs observed for that x axis. The distribution expected according to a binomial distribution is also shown (see text for further explanations).

Despite that TFAM has a low success at recognizing the precise cognate, even for sense tRNAs, analyzing statistically TFAM's output shows it did better than chance. Hence this output can still be informative. The aminoacylation potential for the cognate predicted by the anticodon, as estimated from alignment quality, was greater than that of more than 50% of the 19 non-cognates in 15 sense and 13 antisense mitochondrial tRNAs. Hence 68% of the sense and 62% of the antisense tRNAs have cognate aminoacylation potentials greater than that of half of the remaining 19 non-cognate amino acids (for *E. coli*, this was 96% for sense and 63% for antisense tRNAs). This suggests that on average, antisense tRNAs resemble more sense tRNAs with the same cognate than tRNAs that have other cognate amino acids. This suggests that antisense tRNAs as a group follow mainly the extension hypothesis: their sequences tend to be adapted for correct recognition by tRNA synthetases, at least more than chance. Misacylation potentials (expressed by the number of non-cognate amino acids with higher acylation potential than the cognate) display a bimodal distribution, similar for sense and antisense tRNAs (Figure 2). Few tRNAs have intermediate misacylation potentials. The two separate modes are biased towards the extremes of the distribution: either a tRNA has low misacylation potential (x-axis close to zero in Figure 2), or it has a very high misacylation potential (x axis close to 19). Hence antisense tRNAs can be considered in a binary, qualitative way: those with low misacylation potential (coded as zero), and those with high misacylation tendencies (coded by unity). The discussion deals in more detail with the puzzle that some tRNAs (both sense and antisense) have apparently very high misacylation potentials.

Statistical tests can be applied to these misacylation potentials in Table 2. Here I assume that the focal tRNA sequence is as likely to align with any of TFAM's reference tRNA sequences as the one that has the same cognate amino acid as the focal tRNA sequence that is analysed. Under that null hypothesis, the binomial distribution predicts that the probability to find a focal tRNA that aligns better with 5 or less reference tRNA sequences with different cognates than the focal tRNA's cognate has a two tailed P that is less than 0.05. This means that for that focal tRNA, the observed level of similarity with the reference tRNA that has the same cognate is unlikely to be due to chance. The same logic works for having more than 15 standard tRNAs aligning better than the reference tRNA with the same cognate. Applying this to data in Table 2, misacylation is statistically significantly less likely than random for 13 sense tRNAs (Cys, Phe, Gly, His, Lys, Met, Asn, Pro Gln, Arg, Thr, Trp and Val), and for 10 antisense tRNAs (antisenses of tRNA Ala, Asp, Glu, Phe, His, Met, Pro, Ser UCN, Thr, and Val). Assuming P = 0.05, for 22 tests (meaning 22 tRNAs), 1.1 cases are expected significant at P = 0.05 by chance. Hence many more tRNAs are less misacylated than expected by chance. Using the same principles, 4 sense and 6 antisense tRNAs (sense tRNAs Leu UUR, Leu CUN, Ser AGY and Ser UCN; and the antisense of tRNAs Arg, Cys, Gln, Gly, Leu CUN and Tyr) are more likely to be misacylated than expected by chance. Hence the distribution of misacylation potentials differs highly from that expected by chance, with only 4 sense and 7 antisense tRNAs with misacylation potentials that can be considered as intermediate. Two tailed tests are considered here because it is of interest to see if TFAM's output differs from chance, in relation to both the extension (better than chance) and the avoidance hypothesis (worse than chance).

When more than one statistical test is done, one has to correct P values in a way that takes into account the number of tests done. The Bonferroni correction does this, but is overconservative [54]: it has a high chance of accepting the null hypothesis that there is no effect, even when there is one. Nevertheless, according to that correction, 10 sense and 6 antisense tRNAs still align better than chance with the standard sequence that has the cognate predicted for the focal tRNA by tRNAscan-SE. Using the less overconservative Benjamini-Hochberg method [55] to take into account multiple tests, 13 sense and 10 antisense tRNAs align better than expected by chance at P < 0.05 with the reference tRNA that has the same cognate. Note that whatever method is used, several tRNAs, including sense tRNAs, appear according to TFAM's output as having high misacylation potential. If this was true, it would result in numerous misinsertions during protein synthesis, which is unlikely. This result sheds suspicions towards the biological meaningfulness of TFAM's output. Analyses in the next section show that TFAM estimates are biologically meaningful, while a section in the discussion indicates a possible solution to this apparent puzzle about tRNA misloading and resulting misinsertions.

### A test for robustness of misacylation potentials predicted by TFAM

The following analysis is designed to show that aminoacylation potentials as indicated by TFAM are relatively robust estimates and reflect tRNA biology, despite the apparently counter-intuitive result that TFAM indicates very low aminoacylation specificity, even for some sense tRNAs. These analyses confirm that TFAM is a powerful, though imperfect tool for determining cognate amino acids, even for mitochondrial tRNAs, including their antisense tRNAs.

The anticodons detected by tRNAscan-SE for antisense tRNAs are the inverse complement of the sense tRNA anticodon in the wide majority of cases. In about 5% of all tRNAs examined for the 49 primate mitochondrial genomes used here, the antisense anticodon was not the inverse complement of the sense anticodon. (This excludes tRNA Ser AGY which lacks a D-arm and is not detected by tRNAscan-SE, and tRNAs Leu CUN and Leu UUR for which anticodon symmetry predicts an antitermination anticodon, an anticodon matching a stop codon). In these antisense tRNAs lacking anticodon symmetry, the antisense tRNA has two predicted cognate amino acids: the cognate predicted by anticodon symmetry which is observed in most homologous tRNAs from closely related species, and the anticodon predicted by tRNAscan-SE's analysis of the structure of the antisense tRNA's anticodon arm in that specific species. As stated above, both methods yield the same prediction in about 95% of the cases, but the remaining 5% can be used to test whether TFAM produces robust predictions of aminoacylation potential. Because the 49 primate genomes produce only 40 such antisense tRNAs lacking anticodon symmetry, I expanded the sample by analyzing 76 additional mammalian mitochondrial genomes (Cetacea, 25; Insectivora, 11; Pinnipedia, 23; and Rodentia, 17, which were all the genomes available for these taxa in Genbank in spring 2009). Their tRNA sequences were available because they had been extracted in relation to results described in [46]. This yields a total of 130 antisense tRNAs lacking anticodon symmetry. For each of these antisense tRNAs, I performed two separate analyses of TFAM's output, using as predicted cognate amino acid the one predicted by tRNAscan-SE and the one predicted by anticodon symmetry. Then I compared the misacylation potentials obtained assuming the symmetry- versus the structure-based cognate amino acid predictions. If TFAM's output is relatively robust, it should yield lower misacylation potentials for cognates predicted by tRNAscan-SE than by anticodon symmetry, and this despite that the tRNA sequence can not have evolved much as compared to closely related species where that sense-antisense tRNA has anticodon symmetry. For anticodons predicted by symmetry, on average 10.5 ± 6.8 non-cognates have a greater aminoacylation potential than the predicted cognate amino acid according to TFAM. For anticodons predicted according to the anticodon arm's structure, fewer (9.1 ± 6.1) non-cognates have a greater aminoacylation potential than the predicted cognate amino acid according to TFAM. This difference is small, but a paired t-test shows that it is statistically significant (t = 1.91, one tailed P = 0.029). This result is also confirmed if using on the same data the more robust, non-parametric Wilcoxon test for differences between two medians (z = 1.86, one tailed P = 0.031) (statistical properties of the alignment scores are not known and it is possible that assumptions of normality are inadequate).

The reason for using one tailed tests is that one expects that the 'real' anticodon is the one detected using the specific structure of the tRNA's cloverleaf. The fact that this anticodon only rarely differs from the one predicted by anticodon symmetry means that in some rare cases, the tRNA sequence evolved in a species in such a way that the cloverleaf structure formed by the antisense tRNA differs from the one predicted by pure symmetry from the sense tRNA, yielding an antisense anticodon that is not 'symmetric' with the sense anticodon. If antisense tRNAs are functional and estimates of their aminoacylation potentials by TFAM are valid, then the misacylation potential according to anticodon symmetry has to be greater than the misacylation potential for the cognate predicted by tRNAscan-SE.

Because the identity of the antisense anticodon differs in specific species from that found in almost all other species in that group, the results suggest that upon the change in antisense anticodon from the usual antisense anticodon, the signals in the antisense tRNA's sequence that determine its aminoacylation potential evolved so as to match better the new anticodon. This result means that the aminoacylation potential as determined by TFAM coevolves with the identity of the antisense anticodon, even at the level of micro-evolution between relatively closely related species within a mammalian taxon. This result shows that predictions of aminoacylation potentials by TFAM, though imperfect, are quite robust and are biologically meaningful.

### Sequence complementarity does not predict misacylation

Sense and antisense tRNAs are by definition complementary. Hence many of the properties of the antisense tRNA might seem adaptive not because of function at the level of the antisense tRNA, but because the sense tRNA adapted to translation, and the antisense inherits part of this by symmetry between complementing sequences. This is not the case for the estimates of misacylation in Table 2. There is almost no correlation between the number of non-cognates with greater acylation potentials than the cognate for the sense tRNA and the corresponding number for its antisense tRNA (r = 0.18). However, it will be important to take into account this issue of sequence complementarity between sense and antisense tRNAs when exploring other antisense tRNA properties. For example, for the structural component of the COVE index output by tRNAscan-SE estimating the cloverleaf formation (see corresponding columns in Table 1), sense-antisense COVEs correlate positively (r = 0.397, one tailed P = 0.037). This correlation is also found in most cases where correlations involve homologous tRNAs from different primate species (column marked as S-A: COVE in Table 3): sense and antisense COVE are positively correlated in 16 among 22 tRNA species. This is particularly strong in unprocessed tRNAs. For misacylation potentials (column marked as S-A: TFAM in Table 3), no general tendency appears. However, for both COVE and misacylation potential, there is an overall tendency for more negative correlations in processed than unprocessed antisense tRNAs. Negative correlations can be interpreted as reflecting, at the level of evolution of tRNA sequences in primates, tradeoffs between sense and antisense tRNAs. Such tradeoffs implicitly indicate that processed antisense tRNAs are functional, and therefore some balance exists between their functional requirements (in terms of cloverleaf formation and acylation potential) and those, for the same properties, of their corresponding sense tRNA.

**Table 3:** Correlations between misacylation potentials and stabilities of cloverleaf structures of tRNAs from 49 primate genomes.

tRNA										S-A	S-A	Sense
Processed	n	r	P	r, res	P	rs	p	rs, res	P	COVE	tfam	r
Ala	39	-0.157	0.171	0.144	0.191	0.208	0.102	0.208	0.149	-0.12	**-0.46**	*0.34*
Asp	44	**-0.348**	0.01*	**-0.283**	**0.032**	**-0.4**	0.004*	**-0.4**	0.009*	0.18	-0.07	0.04
Gln	36	-0.028	0.436	0.017	0.461	*0.287*	***0.045***	*0.287*	***0.017***	-0.12	-0.03	0.12
Glu	44	-0.096	0.268	-0.046	0.383	0.023	0.442	0.023	0.332	0.19	0.13	-0.09
Ile	48	0.079	0.299	-0.182	0.111	-0.033	0.413	-0.033	0.19	-0.26	-0.04	**-0.49$**
Met	49	-0.105	0.237	-0.133	0.182	-0.13	0.187	-0.13	0.33	-0.14	-0.16	0.24
Pro	43	**-0.356**	**0.009$**	**-0.417**	**0.007$**	**-0.338**	**0.013**	**-0.338**	**0.017**	**0.41$**	0.22	0.29
Ser UCN	29	-0.106	0.292	-0.112	0.282	-0.077	0.345	-0.077	0.353	-0.03	**-0.32**	-0.01
Thr	34	-0.057	0.375	-0.016	0.464	-0.052	0.385	-0.052	0.492	0.15	0.21	0.09
Trp	49	0.176	0.113	0.182	0.105	0.179	0.109	0.179	0.057	-0.34	0.07	-0.10
Tyr	37	**-0.591**	**0.0001$**	**-0.589**	**0.0001$**	**-0.587**	**0.0001$**	**-0.587**	**0.0001$**	**0.30**	-0.25	**-0.36**
Unprocessed												
Arg	49	-0.11	0.227	-0.048	0.37	-0.073	0.308	-0.073	0.4	**0.38$**	-0.18	0.26
Asn	49	**-0.387**	**0.003$**	**-0.308**	**0.015**	**-0.354**	**0.005**	**-0.354**	**0.02**	0.07	**-0.33**	0.23
Cys	48	0.091	0.268	0.018	0.451	-0.094	0.263	-0.094	0.148	0.26	**0.28**	**-0.29**
Gly	46	0.027	0.429	0.063	0.339	0.044	0.387	0.044	0.377	**0.27**	-0.24	0.12
His	48	0.027	0.427	0.032	0.416	-0.031	0.418	-0.031	0.433	0.04	0.02	0.1
Lys	20	0.093	0.349	0.084	0.363	0.126	0.298	0.126	0.158	0.22	0.13	-0.09
Phe	44	-0.143	0.178	-0.208	0.088	-0.214	0.081	-0.214	0.096	**0.27**	-0.12	-0.28
Val	46	0.023	0.441	-0.065	0.334	0.165	0.137	0.165	0.304	0.24	0.10	-0.2
Leu CUN	6	*0.84*	*0.018*	0.3	0.282	0.235	0.327	0.235	0.5	**0.49$**	n.d.	*0.47$*
Leu UUR	18	-0.059	0.407	-0.28	0.13	0.09	0.361	0.09	0.214	**0.38$**	0.12	0.18
Ser AGY	49	-0.029	0.42	-0.122	0.199	-0.062	0.335	-0.062	0.171	0.01	0.04	0.17
		χ2		χ2		χ2		χ2				
Processed		51.82	0.0003	55.56	0.00009	52.75	0.0003	48.23	0.0010			
Unprocessed		37.60	0.0203	39.48	0.01243	34.69	0.0417	33.28	0.0581			
All		89.42	0.00006	95.04	0.00001	87.45	0.0001	81.51	0.0005			

Correlations are for homologous tRNAs from 49 primate genomes, but exclude antisense tRNAs with non-symmetric anticodons (see text for explanations). Misacylation potentials are deduced from TFAM's output, as shown for human mitochondrial tRNAs in Table 2. The structural component of the COVE index from tRNAscan-SE estimates cloverleaf stability. For antisense tRNAs, residual COVE is the residual from the regression of antisense COVE with corresponding sense COVE. Both parametric Pearson (r) and non-parametric Spearman rank (rs) correlation coefficients and their statistical one sided significances are indicated for antisense tRNAs. S-A indicates parametric correlations between properties of sense and antisense tRNAs (COVE for correlations between cloverleaf stabilities, TFAM for correlations between misacylation potentials). Bold indicates statistical significance at P < 0.05 according to the working hypothesis. $ indicates that the correlation is significant at P < 0.05 after Bonferroni's adjustment of P's for multiple tests, asterisks indicate P < 0.05 after adjusting according to the Benjamini-Hochberg method, but not Bonferroni's. The last three rows indicate the statistics and P values after combining tests in that column according to Fisher's method for combining P values, separately for processed and unprocessed antisense tRNAs, and for all tRNAs.

### Processed antisense tRNAs have higher cognate specificity than unprocessed ones

Previous sections described properties of sense and antisense tRNAs, such as the stability of the cloverleaf secondary structure they form, their predicted cognate amino acid, the potential for misacylation, and whether they are processed or not by sense RNA maturation. The next sections explore whether different properties important for tRNA function correlate in the way coevolution for antisense tRNA function would expect (i.e. one expects cloverleaf stability to correlate negatively with misacylation potential). When possible, the pair of properties is analysed by comparing homologous tRNAs from 49 different primate genomes.

The working hypotheses predict that antisense tRNAs with low misacylation potentials should expand the tRNA pool and hence should be processed by sense RNA maturation, while translational activity by those with high misacylation potentials should be avoided because causing amino acid misinsertions in protein sequences. Hence such antisense tRNAs should be more common among antisense tRNAs that are unprocessed by sense RNA maturation. This test can not be done when comparing homologous tRNAs, because no variation in presence/absence of processing exists within homologous primate tRNAs. Therefore non-homologous tRNAs are compared here. I present results only for the mt genome of *Homo sapiens*. A t-test can be used for misacylation potentials. For mitochondrial human antisense tRNAs that are not processed by sense RNA maturation, the mean misacylation potential = 11.7 ± 6.27. If the tRNA sequence was random, one would expect better alignments than with its expected tRNA homologue for half the tRNAs with non-cognates, hence 9.5 among 19. The reported mean does not differ significantly from 9.5, the number of non-cognates with acylation scores higher than the cognate expected under random conditions. The average for processed antisense tRNAs is 5.36 ± 6.17. This value does differ significantly from 9.5, the expected value: t = -2.22, P = 0.025 (one tailed test). This result specifically supports the extension hypothesis for processed antisense tRNAs, and overall the extension-avoidance hypothesis for all antisense tRNAs: processed and unprocessed antisense tRNAs significantly differ in their tendencies for misacylation (t = 2.33, one tailed P = 0.0155).

Hence antisense tRNAs that are processed during normal sense RNA maturation are better adapted for translation than unprocessed ones because they are less misloaded, which indicates coadaptation between their anticodon and the parts of the tRNA that are recognized by tRNA synthetases.

### Misacylated antisense tRNAs form unstable cloverleaves

Cloverleaf stability is one of the most important properties of tRNAs. For example, tRNA sequences with pathogenic mutations typically form less stable cloverleaves than non-pathogenic variants [56]. Hence one can expect that antisense tRNAs with high misacylation potential form less stable cloverleaves than those with low misacylation potential if the working hypothesis is valid. However, because of their complementarity, secondary structure formation by antisense tRNAs is correlated with that of their sense complement. Hence in order to test for effects on antisense tRNA COVE without these being confounded by the correlation between sense and antisense cloverleaf COVES, I calculated residual antisense tRNA COVE from the linear regression between antisense tRNA COVE as dependent and sense tRNA COVE as independent. These residuals estimate antisense tRNA COVEs independently of their sense tRNA counterpart's COVE: a positive residual means the antisense COVE is greater than would be expected according to the COVE of its sense counterpart, and vice versa for negative values.

The working hypothesis predicts that misacylated antisense tRNAs should form less stable cloverleaves. I tested for correlation between misacylation and cloverleaf stability using COVE of antisense tRNAs, but also using residual antisense tRNA COVE.

**Figure 3:**
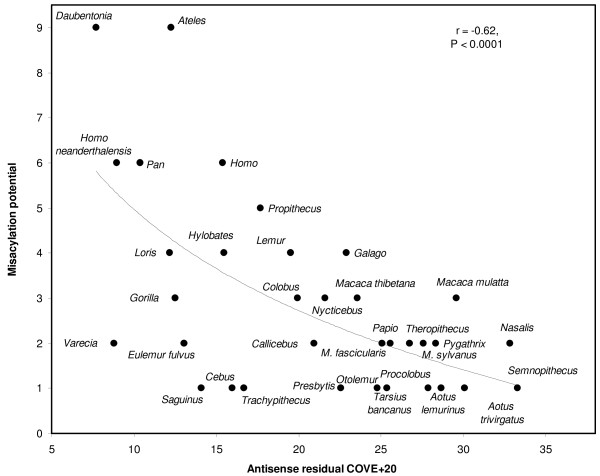
**Misacylation potential as a function of antisense tRNA cloverleaf stability for the antisense of tRNA Tyr**. The y axis is the number of amino acids with greater aminoacylation potential than serine, which is the predicted cognate for that antisense tRNA. The x axis is the residual of antisense COVE, from the linear regression between antisense COVE (dependent) with sense COVE (independent). Only taxa where sense-anticodon symmetry exists are considered, species are followed by NCBI accession numbers: *Aotus lemurinus*, FJ 85421; *A. trivirgatus*, AY 250707; *Ateles belzebuth*, FJ 785421; *Callicebus donacophilus*, FJ 785423; *Cebus albifrons*, NC 002763; *Colobus guereza*, NC 006901; *Daubentonia madagascariensis*, NC 010299; *Eulemur fulvus*, NC 012766; *Eulemur mayottenis*, NC 012769; *Galago senegalensis*, NC 012761; *Gorilla gorilla*, NC 001645; *G. gorilla gorilla*, NC 011120; *Homo sapiens*, NC 012920; *Homo neanderthalensis*, NC 011137; *Hylobates lar*, NC 002082; *Lemur catta*, NC 004025; *Loris tardigradus*, NC 012763; *Macaca fascicularis*, NC 012670; *M. mulatta*, NC 005943; *M. sylvanus*, NC 002764; *M. thibetana*, NC 011519; *Nasalis larvatus*, NC 008216; *Nycticebus coucang*, NC 002765; *Otolemur crassicaudatus*, NC 012762; *Pan paniscus*, NC 001644; *P. troglodytes*, NC 001643; *Papio hamadryas*, NC 001992; *Pygathrix roxellana*, NC 008218; *Presbytis melalophos*, NC 008217; *Procolobus badius*, NC 009219; *Propithecus coquereli*, NC 011053; *Saguinus oedipus*, FJ 785424; *Semnopithecus entellus*, NC 008215; *Tarsius bancanus*, NC 0021811; *Theropithecus gelada*, FJ 785426; *Trachypithecus obscurus*, NC 006900; *Varecia varecia*, NC 012773. Species explored but not included because of lack of anticodon symmetry in that specific tRNA are: *Chlorocebus aethiops*, NC 007009; *C. pygerythrus*, NC 009747; *C. sabaeus*, NC 008066; *C. tantalus*, NC 009748; *Eulemur macaco*, NC 012771; *E. mongoz*, NC 010300; *Perodicticus potto*, NC 012764; *Pygathrix nemaeus*, NC 008220; *Pongo abelii*, NC 002083; *Pongo pygmaeus*, NC 001646; *Saimiri sciureus*, NC 012775; *Tarsius syrichta*, NC 012774.

The extension-avoidance hypothesis expects a negative association between antisense tRNA COVE and the misacylation potential for that antisense tRNA. Considering the 49 primate genomes, I analysed for each of the 22 sets of 49 homologous tRNA genes the correlations between misacylation and COVE for sense and antisense tRNAs, and between misacylation and residual COVE for antisense tRNAs. Table 3 presents the correlation coefficients of these analyses. First, for sense tRNAs (last column in Table 3), the correlation between COVE and misacylation is negative, as expected, for 13 among 22 sense tRNAs. Specific correlations were significant at P < 0.05 in 7 cases, among which four were in the expected negative direction and three in the opposite direction. The correlations where misacylation increases with COVE suggest the possibility that in some sense tRNAs, a tradeoff exists between the two properties because they reached some evolutionary optimum, meaning that in order to evolve higher aminoacylation specificity, a tRNA has to decrease its cloverleaf stability, and vice versa. This rationale of evolutionary optimum and tradeoff between acylation specificity and COVE is less likely to exist in antisense tRNAs, where one expects a less stable situation from an evolutionary point of view. Indeed, only for the antisense of tRNA Lys such a tradeoff seems to exist, but this analysis involves only 6 primate species because only in these species anticodon symmetry exists for this sense-antisense tRNA pair. Note that analyses for antisense tRNAs in Table 3 exclude species where sense and antisense anticodons are not symmetric, besides for the antisense tRNAs of Leu CUN and UUR where only the lack of symmetry between anticodons predicts a cognate amino acid for the antisense tRNA. Results were similar when correlating misacylation with the residual COVE of the antisense tRNA, and when correlation tests used the more robust non-parametric Spearman rank correlation coefficient (rs in Table 3), which does not assume normality for COVE and TFAM's scores. Figure 3 shows one of these correlations. It plots the misacylation potential for the antisense of tRNA Tyr as a function of the residual of the COVE of that antisense tRNA, in all 49 primate species. I did meta-analyses of the statistical significances of the correlation coefficients in each column relating to antisense tRNAs, using Fisher's test for combined P values. This test sums over all k tests considered -2*lnPi, where i ranges from 1 to k and P is the statistical significance of the correlation statistic. This sum has a chi square distribution with 2*k degrees of freedom. The meta-analysis of antisense tRNAs shows that for any type of correlation coefficient, results in Table 3 indicate a statistically significant negative correlation between COVE and misacylation. When analysing separately processed and unprocessed antisense tRNAs, results always show less significant results for the unprocessed antisense tRNAs, which also fits with the prediction that processed antisense tRNAs are more likely to expand the tRNA pool than unprocessed ones. Indeed, for the third column in Table 3, r is negative as expected for nine among eleven processed antisense tRNAs, which is significantly more than expected by chance according to a one tailed sign test (P = 0.016) (here a one tailed test is used because the null hypothesis expects a specific direction for the examined association). For unprocessed antisense tRNAs, only five among eleven correlations are negative, which does not differ from 5.5 expected by pure chance. These results are strong evidence that antisense tRNAs, and especially processed ones, frequently function in translation, while translational activity by antisense tRNAs that have high misacylation potentials is avoided by them forming unstable cloverleaves. This result is remarkable also in the light of corresponding analyses for the sense tRNAs. These indicate that there is no trivial reason to expect a specific direction for correlations between misacylation and COVE, and hence serve as control for results of the analyses of antisense tRNAs.

The same principle of association between misacylation and COVE is also tested comparing only the 22 non-homologous human antisense tRNAs. Comparisons between non-homologous genes yield evidence that is usually considered as less strong than comparisons between homologous genes. However, in our context, these non-homologous comparisons are useful because future experiments designed to test the bioinformatic evidence presented here will most likely focus on tRNAs from given model species which probably include *Homo sapiens*. Hence it is important to show, for *Homo*, that the various antisense tRNAs also follow the principles discussed, and to indicate which antisense tRNAs are most likely to be functional. Figure 2 shows that misacylation potentials have a bimodal distribution in *Homo*. Therefore, residual cloverleaf stabilities of antisense tRNAs from each mode in Figure 2 are analyzed separately. Those with low misacylation potential have a mean residual stability of 5.29 ± 10.96, n = 13, and this value is almost significantly greater than zero (t = 1.741, P = 0.0535, one tailed test). Those antisense tRNAs with high misacylation potential have a low mean residual stability of -8.60 ± 9.74, n = 8, which is significantly lower than zero (t = -2.499, one tailed P = 0.0205). The latter test supports the avoidance hypothesis (it is one tailed because the avoidance hypothesis expects that residual COVE is negative), the former the extension hypothesis (again one tailed, because this hypothesis expects positive COVE values), and the fact that these two means differ significantly (t = -2.938, one tailed P = 0.004) supports the extension-avoidance hypothesis. Hence the principles suggested by comparisons of homologous tRNAs from different primates in Table 3 are qualitatively valid at the level of non-homologous comparisons within mitochondria of *Homo sapiens*.

### Misacylation and accuracy of antisense tRNA processing by sense RNA maturation

**Figure 4:**
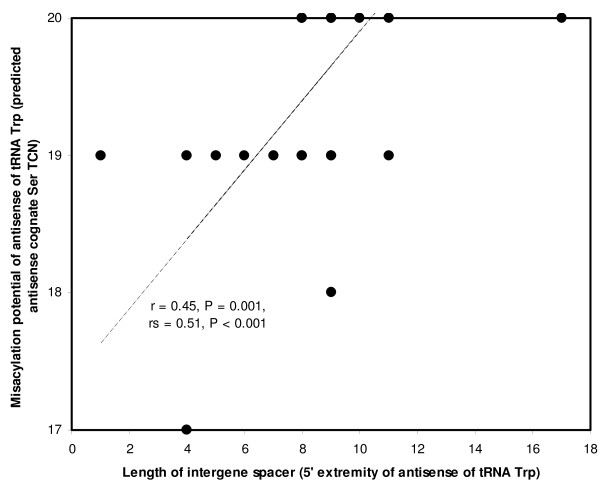
**Misacylation potential of antisense of tRNA Trp as a function of processing inaccuracy of its 5' extremity by sense RNA maturation**. The x axis is the number of nucleotides between the next sense gene (tRNA Ala) and the 5' extremity of the antisense of tRNA Trp. Datapoints are for the same species as in Figure 3, but species excluded for lack of anticodon symmetry between sense and antisense tRNAs differ.

Half the antisense tRNAs have at least one of their extremities processed by sense RNA maturation. This means that an adjacent active sense gene is processed at least at one of their extremities. The number of nucleotides separating the extremity of that adjacent sense gene from the antisense tRNA estimates the lack of precision of processing of the antisense tRNA by sense RNA maturation of that antisense tRNA. One can expect that when antisense tRNAs are processed exactly at their extremity (when there is no intergene spacer), then sense RNA maturation adapted for antisense tRNA translational activity. This is suggested by the observation that most human mitochondrial sense tRNAs have no intergene spacers (not shown). The length of these antisense spacers is indicated in Table 1 (they are summed when both 5' and 3' antisense tRNA extremities are processed). Such intergene spacer lengths were obtained for all 49 primate genomes, and correlations between interspacer length and misacylation were calculated for each of the 11 antisense tRNAs with at least one processed extremity, using the sum of the 5' and 3' spacers in the cases where both extremities are processed. The extension-avoidance hypothesis expects that processing is inaccurate for antisense tRNAs with high misacylation potential, and vice versa for those with high cognate aminoacylation specificity, hence positive correlations are expected between intergene spacer length and misacylation potential, which means that tests described below are one tailed. Figure 4 plots an example, for the antisense of tRNA Trp. Table 4 shows these correlations (using both the parametric Pearson correlation coefficient r and the more robust non-parametric Spearman rank correlation coefficient rs) for all processed antisense tRNAs. A tendency exists for correlations to have the direction that is expected by the working hypothesis. The Benjamini-Hochberg adjustment of the Bonferroni adjustment for multiple tests shows that for at least one (according to r) or two (according to rs) antisense tRNAs, the correlation between misacylation and length of intergene spacer is significant at P < 0.05 after considering that 11 tests were done. Fisher's test for combined P's (last rows in Table 4) also shows that data in Table 4 show a significant tendency for positive correlations, especially for rs.

**Table 4:** Correlations of lengths of intergene spacer between processed antisense tRNAs and the next gene with misacylation potential and cloverleaf stability of antisense tRNAs.

	Misacylation-interspacer				COVE-interspacer				resCOVE-interspacer			
tRNA	r	P	rs	P	r	P	rs	P	r	P	rs	P
Ala	-0.157	0.167	-0.196	0.113	-0.198	0.086	-0.196	0.089	-0.073	0.308	-0.063	0.334
Asp	0.081	0.304	**0.298**	**0.026**	-0.229	0.057	-0.208	0.076	**-0.374**	**0.004$**	**-0.36**	**0.006**
Gln	0.152	0.191	0.18	0.15	*0.36*	*0.006*	*0.331*	*0.01*	*0.314*	*0.014*	*0.288*	*0.022*
Glu	0.144	0.179	0.25	0.053	-0.092	0.264	-0.13	0.187	-0.225	0.06	**-0.239**	**0.049**
Ile	0.047	0.377	-0.135	0.185	0.009	0.477	-0.013	0.464	-0.043	0.384	-0.047	0.374
Met	-0.058	0.346	0.009	0.476	*0.273*	*0.029*	*0.242*	*0.047*	0.119	0.207	0.118	*0.209*
Pro	0.182	0.121	**0.362**	**0.009$**	**-0.412**	**0.002$**	-0.224	0.06	**-0.435**	**0.001$**	**-0.265**	**0.033**
Ser UCN	-0.122	0.256	0.008	0.484	**-0.335**	**0.009**	**-0.324**	**0.012**	-0.04	0.391	-0.032	0.415
Thr	-0.195	0.135	-0.196	0.134	0.099	0.248	0.063	0.334	0.0001	0.498	-0.04	0.392
Trp	**0.45**	**0.001$**	**0.511**	**0.000$**	0.031	0.416	0.034	0.408	-0.009	0.474	-0.001	0.496
Tyr	-0.112	0.254	-0.109	0.26	0.087	0.276	0.155	0.144	0.174	0.116	0.224	0.061
Combined χ^2^		35.78		51.28		48.00		35.86		50.82		33.71
Combined P		0.032		0.0004		0.0011		0.031		0.0005		0.0525

Correlations are for homologous tRNAs from 49 primate genomes, but exclude antisense tRNAs with non-symmetric anticodons (see text for explanations). Misacylation, COVE and residual COVE are as in Table 3. Both parametric Pearson (r) and non-parametric Spearman rank (rs) correlation coefficients and their statistical one sided significances are indicated. The two last rows indicate results of Fisher's method for combining P values. Bold indicates P < 0.05, $ indicates statistical significance at P < 0.05 after Bonferroni's adjustment for multiple tests.

This result is also confirmed at the level of comparisons between non-homologous antisense tRNAs from the human mitochondrial genome. As stated in the previous section, this result is weaker evidence than correlations between homologous tRNAs, but it confirms the principle at the level of a single genome, which is particularly convenient for experimental tests of the hypothesis presented here, because such tests will probably focus on a single genome. For non-homologous human antisense tRNAs, misacylation increases with intergene spacer length (Pearson's r = 0.598, one tailed P = 0.026, n = 11). These results on misacylation and interspacer length are strong evidence for the extension-avoidance hypotheses: the organization of mitochondrial genomes seems finely tuned for long intergene spacers to down-regulate the expression of antisense tRNAs that are likely to be misacylated, but who are necessarily processed by sense RNA maturation because of their vicinity with sense genes.

### Cloverleaf stability and accuracy of antisense tRNA processing by sense RNA maturation

**Figure 5:**
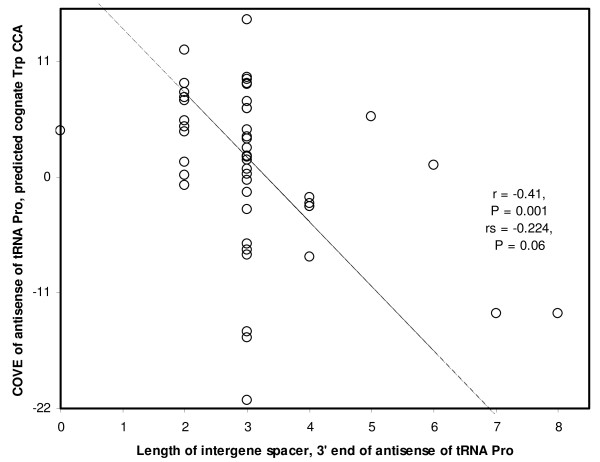
**Cloverleaf stability of antisense of tRNA Pro as a function of the accuracy of processing of its 3' extremity by sense RNA maturation**. The x axis is the number of nucleotides between the next sense gene (tRNA Thr) and the 3' extremity of the antisense of tRNA Pro. Datapoints are for the same species as in Figure 3, but species excluded for lack of anticodon symmetry between sense and antisense tRNAs differ.

Similarly to the previous section, here I explore whether, among processed antisense tRNAs, those accurately processed by sense RNA maturation are also more adequate for translation, but now according to the stability of their cloverleaf structure. I test this using the COVE of antisense tRNAs and the residual COVE, which takes into account the effects of the stability of the sense tRNA cloverleaf on the stability of the cloverleaf of its antisense tRNAs. Figure 5 shows an example, for the antisense of tRNA Pro. Table 4 shows these correlations for all 11 processed antisense tRNAs, using parametric and non-parametric tests (r and rs correlation coefficients, respectively). Here, the working hypothesis expects negative correlations, hence one tailed tests are used. This is the case for the majority of antisense tRNAs only when using rs. The number of correlations that are significant in either direction is somewhat balanced, although there are usually more negative correlations. Hence a meta-analysis approach was used to determine whether a general tendency exists in these data. Fisher's test for combined P's shows what the general tendency in the data is an overall significant negative correlation (last rows of Table 4). This evidence is positive, yet weaker than for previous pairs of antisense tRNA properties.

The analysis considering the non-homologous antisense tRNAs in human mitochondria yields a qualitatively similar, yet statistically not significant result, and this only when using residual COVE (r = -0.207, P = 0.27).

The fact that some positive correlations in Table 4 between length of intergene spacer and COVE are sufficiently strong to be detected as statistically significant suggests that for these two properties, associations may be determined sometimes according to a more complex rationale than the one proposed until now. Indeed, tRNA processing depends on the tRNA's capacity to form secondary structure. It is possible that for processed antisense tRNAs that are usually expressed, a strong cloverleaf structure suggests a reaction to the need for being processed independently of sense RNA maturation, but on the sole 'merit' of the antisense tRNA's own capacity to form secondary structure. If this is correct, inaccurate sense RNA maturation as estimated by long intergene spacers should be compensated by greater cloverleaf stability of the antisense tRNA. This seems to be the case for at least one antisense tRNA, the antisense of tRNA Gln. Hence strong correlations between intergene spacer lengths and cloverleaf stability, whatever is their direction, suggest translational activity for antisense tRNAs.

### Antisense tRNA properties coevolve with amino acid usage

This section explores correlations between the antisense tRNA properties described in previous sections (misacylation, cloverleaf stability and processing accuracy) and the usage of their predicted cognate amino acid in the mitochondrial genome. These analyses assume that variation in amino acid usage among primate species should be matched by corresponding variation in the (antisense) tRNA's properties that would make it more adapted for translational activity. Hence one expects negative correlations between an antisense tRNA's misacylation potential and the genome-wide usage of its cognate amino acid (one tailed tests are used because a specific direction is expected). The direction of the correlation is as expected in less than half the cases, for both parametric and non-parametric correlation analyses (Table 5, r and rs, respectively), but most significant correlations, for r and rs, respectively, have the expected direction (3 and 5 cases), while only 2 significant correlations have the opposite direction. Following the same rationale, COVE (and residual COVE) should correlate positively with amino acid usage. In this case, the direction of more than half the cases is according to prediction for each r and rs. Most statistically significant correlations (7 and 5, for r and rs, respectively) are positive, while only 2 correlations with opposite direction were statistically significant (see Table 5). The length of intergene spacers is expected to correlate negatively with amino acid usage, and indeed most correlations are negative, and the only case that is statistically significant, for the antisense of tRNA Ala, is among them. Overall, considering correlations of cognate usage with all antisense tRNA variables, most statistically significant correlations have the direction expected if antisense tRNAs are functional in translation: 10 according to prediction (excluding residuals of COVE to avoid pseudoreplication with analyses on COVE), versus 3 significant cases that have directions opposite to predictions. Data in Table 5 indicate no significant tendency for processed antisense tRNAs to fit better predictions than unprocessed ones. Results are for evolutionary changes in amino acid usages, suggesting that the antisense tRNAs function in translation.

**Table 5:** Correlations of genome-wide amino acid usages with misacylation potential, cloverleaf stability and processing inaccuracy of antisense tRNAs predicted to be loaded by that amino acid.

tRNA		TFAM		COVE		resCOVE		Spacer	
Processed	n	r	rs	r	rs	r	rs	r	rs
Ala	39	**-0.29**	**-0.32**	**0.29**	**0.38**	**0.28**	**0.35**	**-0.37**	**-0.47**
Asp	44	0.01	0.02	0.23	0.23	0.23	0.23	-0.15	-0.12
Gln	36	**-0.30**	**-0.46**	**0.50**	**0.52**	**0.51**	**0.55**	-0.02	-0.11
Glu	44	-0.14	**-0.25**	-0.08	-0.05	-0.12	-0.11	-0.10	-0.10
Ile	48	0.02	-0.04	0.04	0.09	-0.13	-0.05	0.04	0.01
Met	49	0.09	-0.16	-0.16	-0.21	-0.14	-0.12	-0.16	-0.14
Pro	43	**-0.26**	**-0.28**	0.04	0.12	0.01	0.13	0.10	0.11
Ser UCN	29	0.26	0.19	0.18	0.23	0.17	0.21	-0.19	-0.20
Thr	34	0.24	0.21	-0.11	-0.04	-0.09	-0.17	0.04	0.22
Trp	49	-0.19	0.17	**-0.52**	**-0.56**	**-0.53**	**-0.54**	0.04	0.16
Tyr	37	-0.04	-0.22	**-0.39**	**-0.41**	**-0.37**	**-0.40**	0.05	0.13
Unprocessed									
Arg	49	0.17	0.20	0.14	0.17	-0.10	-0.04		
Asn	49	0.22	0.17	0.06	0.10	0.11	0.14		
Cys	48	**0.31**	**0.29**	**0.34**	**0.33**	**0.33**	**0.35**		
Gly	46	-0.05	0.02	0.13	0.08	0.14	0.13		
His	48	0.01	0.02	**0.29**	0.24	**0.30**	**0.25**		
Lys	20	0.11	0.07	**0.42**	**0.39**	**0.38**	0.30		
Phe	44	-0.24	-0.13	**0.33**	**0.34**	**0.29**	**0.32**		
Val	46	0.05	-0.01	0.05	-0.02	0.07	-0.03		
Leu CUN	6	0.29	-0.22	-0.69	-0.73	-0.65	-0.64		
Ser AGY	49	**0.43**	**0.40**	**0.33**	0.24	**0.33**	0.24		
									
% as expected		36	46	68	64	59	55	55	55

The extension hypothesis expects negative correlations for misacylation and processing inaccuracy, and positive ones between cloverleaf stability (COVE) and amino acid usage. The last row indicates the percentage of correlations whose directions match predictions.

### Summarizing results for associations between antisense tRNA properties

The above sections present each of the properties of antisense tRNAs used to test whether these tRNAs adapted for translation, and pairwise correlations between these properties, and with genome-wide amino acid usage. Overall, the hypothesis that antisense tRNAs function in translation yields predictions for the directions of each of the 6 pairwise combinations of these 4 antisense properties. Similarly, the working hypothesis also predicts the direction of correlations between these properties and amino acid usage, hence in total 9 pairwise combinations.

Table 6 summarizes the results of tests of the extension-avoidance hypothesis presented here for pairs of properties. Results are similar when considering parametric and non-parametric analyses, and indicate the percentage of tRNAs for which the direction of the correlation was according to the prediction for that pair of variables. For processed versus unprocessed antisense tRNAs, the table presents the percentages for the 11 processed ones followed by the percentage among the unprocessed ones. For COVE (indicated by stability in Table 5), percentages for COVE and the residuals of COVE are indicated. The majority of the tRNAs follow the predictions of the working hypothesis, and this for the majority of pairiwise variable combinations. This is also true when comparing processed versus unprocessed antisense tRNAs, as the working hypothesis expects more cases fitting the hypothesis among processed than unprocessed antisense tRNAs. The fact that these various analyses are not independent of each other makes it difficult to assess the statistical significance of the results, but the general tendency is clear.

**Table 6:** Percentages of antisense tRNAs for which correlations fit expectations according to the hypothesis that antisense tRNAs are active in translation, for all pairs of variables examined in Tables 3-5, separately for parametric and non-parametric correlation tests.

		Parametric	Nonparametric
Misacylation			
	COVE	68*	55
	resCOVE	64	64
	Spacer	55	64
	Usage	38	48
COVE Res COVE			
	Spacer	46	46
		64	73*
	Usage	67	62
		57	52
Spacer	Usage	55	55
			
Processing	Misacylation		82*/46
	COVE		82*/82*
	res COVE		55/55
	Mis-COVE	82*/46	64/55
	Mis-res COVE	73*/55	64/55
	Mis-Usage	55/20	64/30
	COVE-Usage	55/90*	55/80*
	Res COVE-Usage	46/80*	46/70

For correlations between COVE, the index for capacities to form cloverleaf structures (from tRNAscan SE [37]), and other variables, the first row indicates results using antisense tRNA COVE scores, the second row indicates results for residuals of antisense tRNA COVE scores, calculated from the linear regression between antisense tRNA COVE (dependent) with the COVE score of its corresponding sense tRNA (independent). * indicate that the percentage is significantly greater at P < 0.05 than 50% according to a sign test. The variable 'processing' indicates the presence or absence of antisense tRNA processing by sense RNA maturation. For processing, the first percentage is for processed antisense tRNAs, the second for unprocessed ones. The working hypothesis expects higher percentages for processed than unprocessed antisense tRNAs.

Note that data in Table 3 suggest that the correlations between COVE and misacylation, and residual COVE and misacylation fit less well predictions for unprocessed than processed antisense tRNAs (compare P values for processed and unprocessed antisense tRNAs in Table 3). Hence presence or absence of processing also interacts with the strength of associations between other properties important for antisense tRNA function. Similarly, associations between sense and antisense COVE, and sense and antisense misacylation potentials suggest differences between processed and unprocessed antisense tRNAs. The results, in that case, support the idea that for processed antisense tRNAs, which are according to the working hypothesis the most likely to be functional, tradeoffs might exist between the property of the sense and the antisense tRNA, while no indication for such tradeoffs exists for unprocessed antisense tRNAs. The overall results of tests done for any combination of tRNA properties for homologous tRNAs support the extension-avoidance hypothesis for translational activity by antisense tRNAs (avoided for antisense tRNAs with low COVE, high misacylation potential and long intergene spacer; but promoted for those with high COVE, low misacylation potential and short (or no) intergene spacers).

## Discussion

Results show that on average, some antisense tRNAs are more adapted for translation than others. Both sense and antisense tRNAs are less likely to be misacylated than would be expected if this was random. Hence on average, antisense tRNAs cause less amino acid misinsertions than one would expect if there was no adaptation for them to function in translation. This is particularly the case for those processed by regular sense RNA maturation, as compared to unprocessed ones. They are less likely to be misacylated (Table 2). Antisense tRNAs with high misacylation potentials form weaker cloverleaves than those with low misacylation potentials (Table 3, also Figure 3), and, when they are processed by sense RNA maturation, they tend to be less accurately processed (Table 4, Figure 4).

Assessing the overall significance of the results is not straightforward, because the working hypothesis is tested many times. Correction methods for multiple tests are all somewhat overconservative, but they do indicate that some tests are significant after correction, depending on which criteria are used. The results in Tables 3, 4 &5 as analyzed by methods that take into account multiple testing show the difficulty at assessing in a realistic (rather than conservative) way the significance level of the results. Assuming independence among tRNAs is a simplification that also leads to overconservative interpretation of the data. The use of Fisher's test for combining P values assumes independence, and indicates that results are significant.

### Processing 5', 3' or both extremities

One of the properties of antisense tRNAs analyzed here is whether sense RNA maturation processes the antisense tRNA's extremities. Some antisense tRNAs are processed at their 5', other at their 3' and two at both extremities. Analyses of the associations between other tRNA properties and these different types of processing (5' or 3' extremities) do not yield a clear picture. For example, processing inaccuracy averages at length 5.14 ± 4.10 for intergene spacers next to antisense tRNAs processed at their 5' extremity, and 3.6 ± 2.61 for those not processed at that extremity, which is not statistically significant (t = 0.7, two tailed P = 0.51) and does not fit the prediction that 5' processing is adapted for accuracy. The same test for processing at the 3' extremity yields qualitatively the result that 3' processing is better adapted for antisense tRNA expression than no processing at the 3' extremity, but the result is still weak and not statistically significant (processed, mean intergene spacer length = 3.33 ± 2.42; no 3' processing, mean length = 6.00 ± 4.64, t = 1.23, one tailed P = 0.125). Excluding the two antisense tRNAs that are processed at both extremities does not change qualitatively these results. However, the various associations usually suggest, as in this simple example, that 3' processing is more relevant for antisense tRNA translational activity than 5' processing. This is in line with the peculiarity of mitochondrial sense tRNAs that 5' maturation always occurs before 3' maturation [57]. According to this, one can predict that 3' processing of antisense tRNAs by sense RNA maturation has a higher impact than 5' processing, because it is probably more limiting antisense tRNA production than the faster 5' processing. Hence, for example, the antisense of tRNA Met is more likely to fully mature than the antisense of tRNA Ile, because after normal maturation, only its 5' extremity has to be processed, which is a process more efficient than 3' processing. For the antisense of tRNA Ile, full maturation is hence less probable, because after 5' processing by normal maturation, 3' processing is required, which is a less efficient process.

### tRNA misacylation: competition between tRNA synthetases or between tRNAs?

The quantification of misacylation potential, as deduced from TFAM's output, is central to this study. Note that several results do not depend on this property, but only on cloverleaf stabilities, the presence or absence of processing by sense RNA maturation, the accuracy of that processing, and genome-wide usage of the predicted cognate amino acid. These also yield strong evidence in favor of translational activity by antisense tRNAs. Hence a large part of the evidence for adaptation of antisense tRNAs for translation does not depend on the accuracy and robustness of TFAM's output.

The latter point is important, because for some sense and antisense mitochondrial tRNAs, TFAM predicts very high misacylation potentials. This result seems unrealistic from a biological point of view, because it would yield numerous amino acid misinsertions during protein synthesis if TFAM's output is correct. This point has to be investigated further. The fact that the misacylation potential of even some of the known sense tRNAs is very high suggests that TFAM's results are not very robust for mitochondrial tRNAs. The alternative is that they reflect a biological reality. If the latter case is correct, results depending on TFAM are useful. A section in 'Results' shows that when comparing homologous tRNAs, the variation in TFAM's output can be considered as quite robust. Here I deal with the basic problem that, when comparing non-homologous tRNAs, a sizeable minority of them has a very high misacylation potential.

The classical way of interpreting the output produced by TFAM (see Table 2) is to compare values in the same row. Expressing this in biological terms, this can be interpreted as comparing the affinities of 20 different tRNA synthetases for aminoacylating a given tRNA gene. That approach assumes that the proteins compete among themselves for aminoacylating a tRNA (note that 'competition' is meant in a purely chemical sense). However, in some cases, the opposite might be true: tRNAs compete for aminoacylation by a given tRNA synthetase. This can be also tested, by looking at columns in Table 2 (for simplicity, I consider here only sense tRNAs), and comparing the values across tRNAs, counting the number of times tRNAs with anticodons for amino acids that are not the cognate assumed for that column yield a better (more positive) score than the tRNA with the anticodon that matches the cognate amino acid assumed by that column. For example, for tRNA Ser AGY, for which the row-analysis estimates that serine is the least likely amino acid to be aminoacylated to that tRNA, a column analysis shows that among the 22 human mitochondrial sense tRNAs, tRNA Ser AGY has the highest affinity with the seryl-tRNA synthetase. Hence while assuming competition between tRNA synthetases does not predict correct aminoacylation for tRNA Ser AGY, assuming competition between tRNAs for aminoacylation does predict the correct cognate.

**Figure 6:**
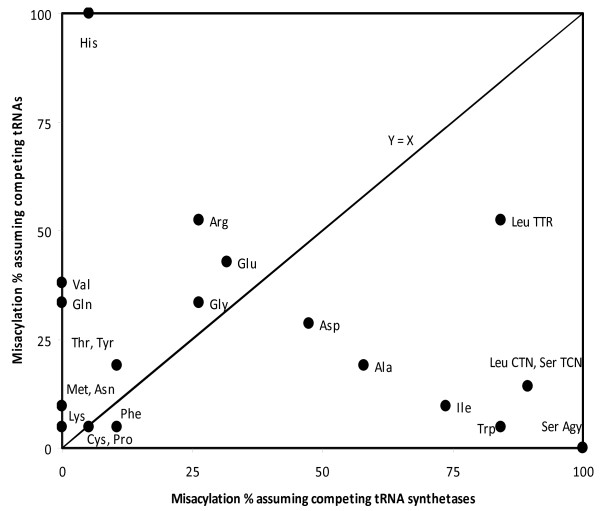
**Column-versus row-analysis of TFAM's output**. The y axis indicates the percentage of non-cognate amino acids with greater amino acylation potential than the cognate according to TFAM, assuming competition among tRNAs for tRNA synthetases (column-analysis of output in Table 2, see text). The x-axis is the percentage of non-cognate amino acids with greater aminoacylation potential than the cognate supposing competition among tRNA synthetases for tRNAs (row-analysis of Table 2). This graph shows that for many sense tRNAs for which classical interpretations of TFAM's output (row analysis of Table 2) yield a poor prediction of the cognate, assuming that tRNAs compete for tRNA synthetases explains apparent high misacylation rates.

Note that the above approach can only be done when all expressed tRNAs are known. I use it here to show that the output from TFAM is in most cases biologically meaningful, even when the classical row analysis yields high misacylation tendencies. For the sake of the example, I use it considering only sense tRNAs. According to this column-analysis of the data in Table 2, the cognate amino acid is better than 50% of the non-cognates for 19 among 22 (86%) sense tRNAs. This is significantly better than chance at P < 0.05 for 14 tRNAs (one tailed tests): Ala, Asn, Cys, Ile, Lys, Leu CUN, Met, Phe, Pro, Ser AGY, Ser UCN, Thr, Tyr and Trp. The column approach improved cognate prediction accuracy upon the row approach in 11 tRNAs, for Ala, Asp, Cys, Ile, Leu CUN, Leu UUR, Phe, Pro, Ser AGY, Ser UCN, and Trp. Figure 6 plots the percentage of amino acids with better aminoacylation potentials than the cognate amino acid according to a column analysis (competition among tRNAs, y axis) as a function of the percentage according to a row analysis (competition among tRNA synthetases, x axis). For 8 among 22 tRNAs (36%), those in the lower left corner of the graph, it does not matter much whether one uses a row- or column-based approach. In these cases, TFAM predicts that the cognate amino acid has among the highest aminoacylation potentials, irrespective of whether one assumes competing tRNA synthetases or competing tRNAs. It is probable that these 8 mitochondrial tRNAs resemble in this respect most other, non-mitochondrial tRNAs, for which TFAM has a high success rate at predicting the cognate. For tRNAs Gln, Val, and especially His, the classical row-approach assuming competition among tRNA synthetases for the tRNA is performing well, but the column-based approach does not. Hence for these 3 tRNAs, aminoacylation is determined by competition among tRNA synthetases for these tRNAs. For a group of 4 or 5 tRNAs (Arg, Asp, Glu, Gly, and maybe Ala), none of the row- and column-based approaches yields a good prediction of the cognate amino acid. Predictions are in both cases intermediate. For this group, aminoacylation might be determined by factors that are not grasped by TFAM's output, or by a combination of row- and column-approaches, meaning assuming a mixture of competition between tRNAs and competition between tRNA synthetases (i.e. tRNAs compete only for tRNA synthetases from their corresponding class). For tRNAs Ile, Leu CUN, Ser AGY, Ser UCN and Trp, results suggest that the row-approach is not appropriate for determining the cognate amino acid, but the column approach is. Hence for these tRNAs, aminoacylation specificity for cognate amino acids seems determined by competition among different tRNAs for the specific tRNA synthetase. For tRNA Leu UUR, both approaches perform badly in predicting the cognate amino acid.

Considering competition between tRNAs suggests that further criteria can be relevant for correct tRNA aminoacylation. It makes sense to suppose that tRNAs with low cloverleaf stabilities are poorer competitors. The weakest structure is formed by tRNA Ser AGY, but, according to TFAM, its aminoacylation potentials by many tRNA synthetases are among the highest, perhaps compensating its low stability. However, because of its low structural stability, and probably even more because its structure is inherently different from other tRNAs (it lacks the D-arm), this tRNA is unlikely to win the competition for tRNA synthetases. This rationale, if adequately used, would probably still improve aminoacylation specificities as estimated by TFAM.

These results on competition between tRNAs for aminoacylation versus competition between tRNA synthetases are worth developing further and probably will yield deeper insights into tRNA aminoacylation by tRNA synthetases. This however is not the topic of this project. These analyses are presented here because they show that the results from TFAM are biologically meaningful, though their interpretation is not as straightforward as one might have believed. The analyses nevertheless show that TFAM's output is not an artifact, also when it predicts high misacylation potential. All analyses in the Results sections use misacylation potentials based on row analyses. Hence they have to be interpreted as misacylation potentials in terms of competition between tRNA synthetases. They are meaningful even if they suggest a low determinism in cognate aminoacylation. One has to keep in mind that this is because only competition between tRNA synthetases is taken into account, and not also between tRNAs. As the latter (column-based) analysis would require a priori knowledge of which tRNAs are expressed and which are not, simple applications of these principles are impractical for antisense tRNAs, and hence inadequate in the context of a project aimed at testing whether antisense tRNAs are expressed.

## General conclusions

The four properties of antisense tRNAs that were explored and are relevant for accurate protein synthesis by such antisense tRNAs (processing, misacylation tendency, cloverleaf stability (also corrected for association with sense tRNA cloverleaf stability) and the accuracy of processing by sense RNA maturation) are on average inter-related in a way that some antisense tRNAs seem adapted for translational activity. This means that these antisense tRNAs could be recruited for translation. These properties also coevolve with genome-wide amino acid usage as one would expect if these antisense tRNAs are active in translation and their evolution reacts to greater usage of their cognate in protein synthesis in some species. The working hypothesis implies that for antisense tRNAs expected according to the above analyses to be routinely recruited in translation, pathogenic mutations, as opposed to nonpathogenic ones, should decrease the tRNA's capacity to function in translation, while for those expected according to the above analyses not to function in translation, pathogenic mutations should increase this capacity. These predictions will be explored elsewhere.

The fact that mitochondrial RNA maturation is largely dependent on tRNAs signalling processing by forming secondary structure makes mitochondrial genomes a good system in which to test first the hypothesis that antisense tRNAs are also sometimes expressed. The results presented here, especially if confirmed by experimental results, indicate the possibility of functional antisense tRNAs in nuclear genomes, and particularly prokaryotes. It is unlikely that sense-antisense coding is restricted to mitochondrial tRNA genes.

## Conclusions

1. Some antisense tRNAs could enrich the pool of mitochondrial tRNAs.

2. Statistically, alignment methods predict sense and antisense tRNA cognate amino acids that match better than expected by chance the amino acid predicted according to the anticodon's identity.

3. The anticodon of the antisense tRNA is usually the complement of the sense tRNA's anticodon. This property is termed sense-antisense anticodon symmetry.

4. In the rare cases where there is no anticodon symmetry, the misacylation potential for the cognate amino acid determined by the non-symmetric antisense anticodon is lower than for the regular, symmetry-determined cognate.

5. Antisense tRNAs processed during normal sense RNA maturation are less misloaded than antisense tRNAs not matured at any extremity, misacylated antisense tRNAs form weaker cloverleaves, processing and acylation accuracies of antisense tRNAs are positively correlated, and these correlate positively with antisense tRNA cloverleaf stabilities.

6. Genome-wide amino acid usage correlates positively with the cloverleaf stability of the antisense tRNAs loaded by that amino acid. Misacylation potentials and processing inaccuracy of antisense tRNAs tend to be inversely correlated with amino acid usage.

7. Low success at detecting cognate amino acids for mitochondrial tRNAs by alignment methods seems due to focusing on aminoacylation specificity as determined by competition among tRNA synthetases for their substrate tRNA. Considering the opposite, that in some cases, specificity results from competition between tRNAs for their tRNA synthetase solves part of this puzzle.

## Competing interests

The author declares that he has no competing interests.

## Authors' contributions

HS is the sole author of this contribution.

## Reviewers' comments

### Reviewer 1: Juergen Brosius

The author proposes that there are more, thus far unrecognized, tRNAs in vertebrate mitochondrial genomes, encoded on the opposite strand of known mitochondrial tRNAs, using a number of parameters including correct secondary structure (how about prediction of three-dimensional structure?) and correct aminoacylation. Going directly to core of the matter, I analyzed some of our own cDNA libraries (unpublished) made from small non-protein coding RNAs (npcRNAs) in an attempt to verify predicted RNAs experimentally. As a control, the bona fide tRNAs shown in Fig. 1 of the submitted manuscript were analyzed as well.

Out of a total of ~80,000 reads from human tissue or cell-line small (60-500 nt) and very small (10-60 nt) RNA libraries, altogether 57 sequences of bona fide mitochondrial tRNA-Asp were identified:

2 sequences with complete 5' end

11 sequences with complete 3' end but without posttranscriptionally added CCA

36 sequences with complete 3' end and with added CCA end

10 sequences close to the 3' end but a few bases missing

The human mitochondrial tRNA-Asp has the following sequence:

LOCUS NC_012920

aaggtattag aaaaaccatt tcataacttt gtcaaagtta aattataggc taaatcctat

atatctta

and is embedded as follows in the human mitochondrial genome (tRNA in bold):

7501 tccatgactt tttcaaaaag gtattagaaa aaccatttca taactttgtc aaagttaaat

**7561 tataggctaa atcctatata tctta**atggc acatgcagcg caagtaggtc tacaagacgc

The predicted sequence of the novel mitochondrial tRNA-Val in antisense is shown below:

>novel Val_Gac_anti-rc

ATATAGGATTTAGCCTATAATTTAACTTTGACAAAGTTATGAAATGGTTTTTC

TAATACC

and how it is embedded in the reverse complement mitochondrial genomic sequence (bold)

GCGTCTTGTA GACCTACTTG CGCTGCATGT GCCATTAAGA T**ATATAGGAT TTAGCCTATA**

**ATTTAACTTT GACAAAGTTA TGAAATGGTT TTTCTAATAC C**TTTTTGAAA AAGTCATGGA

In contrast, from that locus, 4 clones in antisense-orientation were recovered (italics, with predicted tRNA still bold:

mi18P0009G19_R.ab1 (italics) on reverse complement

GCGTCTTGTA GACCTACTTG CGCTGCATGT *GCCATTAAGA T**ATATAGGAT TTAGCCTATA***

***ATTTAACTTT GACAAAGTTA TGAAATGGTT *TTTCTAATAC C**TTTTTGAAA AAGTCATGGA

mi18P0017J15_Rab1 (italics) on reverse complement

GCGTCTTGTA GACCTACTTG CGCTGCATGT *GCCATTAAGA T**ATATAGGAT TTAGCCTATA***

***ATTTAACTTT GACAAAG*TTA TGAAATGGTT TTTCTAATAC C**TTTTTGAAA AAGTCATGGA

mi16P000A17_R.ab1 (italics) on reverse complement

GCGTCTTGTA GACCTACTTG CGCTGCATGT *GCCATTAAGA T**ATATAGGAT TTAGCCTATA***

***ATTTAACTTT GACAAAGTTA *TGAAATGGTT TTTCTAATAC C**TTTTTGAAA AAGTCATGGA

mi18P0019N05_Rab1 (italics) on reverse complement

GCGTCTTGTA GACCTACTTG CGCTGCATGT *GCCATTAAGA T**ATATAGGAT TTAGCCTATA***

***ATTTAACTTT GACAAAG*TTA TGAAATGGTT TTTCTAATAC C**TTTTTGAAA AAGTCATGGA

The first striking observation is that all four clones are extended at the 5' end by 11 nucleotides. In addition, none of the four clones represent the 3' end (with or without posttranscriptionally added CCA end).

The best explanation is that these clones do not represent bona fide tRNAs, but merely processing intermediates that are stable enough to be represented in our cDNA libraries. A possibility that cannot be ruled out at this point is that these RNAs, partially overlapping the predicted tRNAs have a non-tRNA function. Further work on phylogenetic conservation, especially involving secondary structures would be revealing. As an aside, the author often uses the term splicing, where the term processing would be appropriate.

For the > novel Trp_TCA_sense

AAAAAAGGAAGGAATCGAACCCCCCAAAGCTGGTTTCAAGCCAACCCCATGGCCTCCATGACTTTTT

we did not find any clones and two of of the bona fide mitochondrial tRNA-Ser plus about a dozen truncated cDNAs.

Author's reply:

I am grateful for these useful comments, as well as for the efforts invested at examining data according to my hypothesis. The reviewer's comment points out a major flaw in my original manuscript. I neglected to discuss the reasons why antisense tRNAs have not yet been detected, as in the analyses by the reviewer which yield unconvincing or negative results in relation to the antisense tRNA hypothesis.

The hypothesis that antisense tRNAs are functional in regular translation in vertebrate mitochondria follows the rationale of cost minimization. Indeed, because the entirety of both DNA strands is transcribed to RNA, the mitochondrion is more efficient when both sense and antisense tRNAs are active in translation, because this implies less waste of RNA. Therefore, one expects more antisense tRNA activity in organisms that are more selected for fast and efficient growth, development and reproduction, namely r-selected, as opposed to K-selected species.

**Figure 7:**
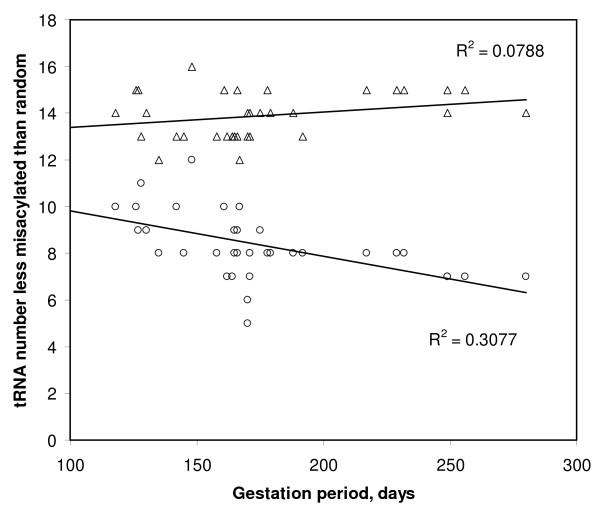
**Tendency for correct amino acylation in sense and antisense tRNAs as a function of duration of gestation in primates**. The y axis plots numbers of tRNAs per mitochondrial genome with less than half non-cognate amino acids with aminoacylation scores (according to TFAM) higher than the tRNA's cognate predicted according to its anticodon. The x axis is the length of the gestation period (days), for primates (gestation data from http://genomics.senescence.info/species/[58]). Triangles: sense tRNAs; Circles, antisense tRNAs. The negative trend for antisense tRNAs suggests that antisense tRNAs are more adapted for translational activity in species with fast development than those with slower development. Data for sense tRNAs are presented as negative control.

**Figure 8:**
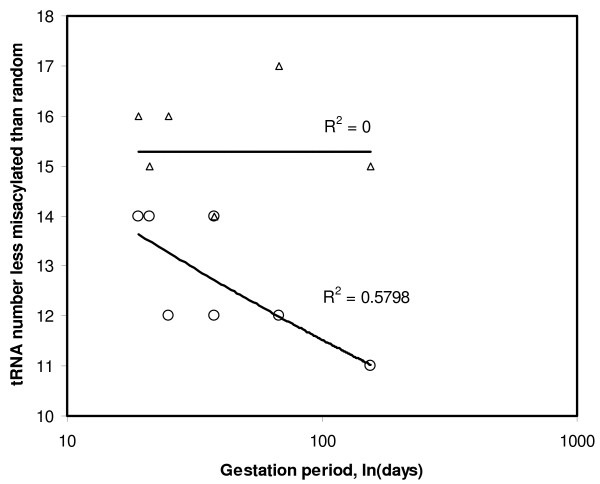
**Tendency for correct amino acylation in sense and antisense tRNAs as a function of duration of gestation in rodents**. The y axis plots numbers of tRNAs per mitochondrial genome with less than half non-cognate amino acids with aminoacylation scores (according to TFAM) higher than the tRNA's cognate predicted according to its anticodon, as a function of the length of the gestation period (days), for rodents (gestation data from http://genomics.senescence.info/species/[58]). Triangles: sense tRNAs; Circles, antisense tRNAs. Results confirm those from Figure 7 for primates and suggest that the association with the duration of gestation is not circumstantial.

In order to test this, I examined correlations between the length of gestation as an indicator inversely proportional to developmental rate (and reflecting the r-K continuum), and a general index of how much antisense tRNAs are adapted to translational activity at mitochondrial genome-wide level in these species. For that purpose, I used the number of antisense tRNAs for which less than half of the non-cognate amino acids have greater aminoacylation potentials with the antisense tRNA than the cognate amino acid, as an estimate of correct aminoacylation of antisense tRNAs in that genome. Figures 7 and 8 plot this number as a function of the length of gestation in primates and rodents, respectively. In both taxa, the correlation for antisense tRNAs is negative and statistically significant at P < 0.05, as expected by the hypothesis that r-selected species (with short gestation) should be more efficient by using also antisense tRNAs: species with fast development have more antisense tRNAs with a tendency for correct aminoacylation than those with slower development. Hence antisense tRNAs seem more adapted for translation, in r-selected species. This also suggests that antisense tRNAs are more expressed under stress conditions, notably hunger and during fast development. The results of reviewer 1 could make sense in this respect: under normal conditions, one can assume that enzymes degrading RNA, such as endoribonucleases, are imported into the mitochondrion from the cytosol, and degrade most RNAs before they become active in translation, besides those RNAs that are recognized as the classical mitochondrial functional RNAs. Under stress, such as hunger, one can expect less import (this process is energetically costy). Therefore, enzyme-mediated degradation should be slower under these conditions, and under such circumstances, antisense tRNAs should be more likely detected. I would therefore suggest that translational activity by antisense tRNAs is more likely to be detected in mitochondria which are under stress, rather than under optimal conditions.

### Reviewer 2: Anthony Poole

This work aims to address the hypothesis that mitochondrial genomes code for antisense tRNAs on the opposite strand from those tRNAs already identified (sense tRNAs). This is an interesting hypothesis, and should certainly be investigated, especially given the fact that a novel mitochondrial small RNA has recently been characterised (Ref 44 - Yu et al. Biochem Biophys Res 372:634). The current manuscript has lots of detailed computational and statistical analyses, but reading it, my major criticism is that the analyses presented are unable to answer the question at hand.

Robust testing of the hypothesis can be done using established experimental procedures to screen for expression of candidate antisense sequences, and whether they are charged. Reference 44, where a previously undetected short mitochondrial RNA was experimentally identified and demonstrated to be charged, shows exactly how such experimental studies can bear fruit.

The barrage of tests attempting to indicate these antisense sequences are functional, and the convoluted arguments as to why tfam can be used for cognate detection (despite obviously poor results) can only be argued to carry any weight if these hypothetical antisense tRNAs can be demonstrated to be expressed. While I do have a number of reservations regarding the analyses presented, these seem minor compared to the need to properly address the question using experiments. Without supporting experimental evidence, the analyses are too weak to be anything more than vaguely circumstantial, and discussing the details of these is therefore academic.

I recommend that the author hold off on attempting to publish this very preliminary work in Biology Direct and instead contact an experimental group with whom he might productively collaborate. Backed up with experimental evidence, some of the analyses the author presents may be more compelling.

Author's reply:

The comments of reviewer 2 are very interesting as these raise the question of how to hierarchise evidence from different nature, in this case statistical patterns versus direct experimental evidence. In my view, the fact that statistical distributions of several antisense tRNA properties tend to follow what one would expect according to the working hypothesis goes beyond the level of circumstantial evidence, because assuming under such circumstances that the working hypothesis is overall correct is most parsimonious. My reply to reviewer 1 suggests that it is the absence of positive molecular evidence that might be circumstantial. This means that direct experimental results are not necessarily more convincing (at least not if they yield negative results) than statistical patterns detected by analyses of bioinformatic data. In any case, the absence of molecular evidence in favour of the working hypothesis does not discredit the relatively positive evidence presented here, even if it is of 'statistical' nature. This is also true for evidence from antisense antiterminator tRNAs [46], which was not yet mentioned in the version of the manuscript examined by the reviewer because it was not yet published. There are reasons why functional antisense tRNAs have not yet been detected (i.e. my reply to reviewer 1), and hence, if these exist, these are not so easy to detect. It is probable that my 'preliminary' analyses are a necessary step to help find the organisms and conditions at which antisense tRNAs are usually active, sparing lots of experimental efforts by making them more efficient, or even worse, leading to the publication of false negative results. Indeed, false positive results are likely to be corrected by ulterior studies repeating analyses. However, false negative results are less likely to be corrected, because less inviting followup studies. For example, in the present case, these studies might have found negative results because mitochondria were naively grown under standard optimal conditions (see my answer to reviewer 1). In the absence of positive results such as those presented here (including Figures 7 and 8), it is probable that preliminary negative results obtained at optimal conditions would have discouraged further experimental enquiries. Publishing positive evidence of the type described here will make little sense after antisense tRNAs are detected by direct experimental methods, but is very useful in the present scenario where such evidence is still lacking.

### Reviewer 3: Andrei S Rodin

The author proposes that mt antisense tRNAs might play an important role in translation, and presents numerous arguments and analyses in support of this notion. Some of them are more compelling than the others; however, they work successfully in the complementary fashion. In general, I found the author's logic, approach and results to be mostly convincing and highly interesting.

Author's reply:

Thank you for the positive comments.

Statistical analysis. Firstly, the author should give a clearer explanation of the choice between the one- and two-tailed tests. Also, I am not sure that the multiple testing correction procedures employed by the author are a good fit in this case. One could argue that *any *such procedure would be too conservative here, not just Bonferroni, because (1) it is likely that the tests are not in fact independent, and (2) we are not exactly sampling from some underlying "true" distribution: what we have a very "closed" system with just so many amino acids and tRNAs. However, this strengthens (rather than weakens) the author's conclusions anyway. Potentially more dangerous is the following: would it be appropriate to interpret a number of strong negative correlations together with a number of strong positive correlations as a bimodal distribution (instead of them simply cancelling each other out)? Finally, using parametric tests with something like "misacylation potential" is debatable, since it is unclear how the variable in question is distributed. Again, this does not really change the author's results/conclusions, but it brings us to another important issue (see below)

Author's reply:

I am very grateful to the reviewer for his insightful comments on the statistical side of my analyses. Indeed, any multiple testing correction procedure is overconservative. I use these in the name of presenting results in a conservative light. The reviewer also understands in depth the complex issues at the core of dependent versus independent multiple tests. I tried to deal with these in the simplest way. I do not have an answer in relation to the bimodal distribution of results, in terms of statistical reduction, besides that bimodal distributions cannot, by essence, be reduced to analyses assuming unimodality. Their existence consists in itself positive evidence. I present for all correlational analyses parametric and non-parametric correlation coefficients, to deal with the potential problem of lack of normality in the distribution of the variables. I believe that parametric statistics are more adequate for estimating an effect (the stronger a correlation, the more we can expect that antisense tRNA to be active in translation), while non-parametric analyses yield more robust, conservative results. By presenting both, I believe that I give a better quantitative description of the phenomenon, while assessing it in relatively robust and conservative terms. Model-based tests, which would combine these advantages, are still less accessible to the average biologists, including myself.

Software and measurements. The author should put at least a brief description of software (tfam, tRNAscan-SE) in the beginning of the manuscript --- what exactly does it do? and how does it do it? What is the biological interpretation of the output (scores, measurements, etc.)? How are these quantities scaled/distributed? The author does have a useful discussion on the robustness, and biological meaning, of some of these in the Discussion section, but by that time it is too late. General Biology Direct audience might be unfamiliar with this type of software and, more importantly, it is somewhat unclear whether the traditional statistical tests (especially parametric ones) are applicable to the variables thus generated.

Author's reply:

I did not find a way to present the softwares earlier in the flow of my text. I hope that the explanations given, which have been expanded in the present version, are sufficient. My view on parametric versus non-parametric tests is given in the previous part of the reply. 

Throughout the manuscript, the author concentrates on four tRNA properties (leading to a series of pairwise analyses) --- however the actual numbers seem to vary from three to six, which is confusing. Perhaps a more explicit list/explanation is in order. The same applies to the "Summarizing results for associations between antisense tRNA properties" section --- why not summarize the results in some sort of an easy-to-grasp cross-table, each cell corresponding to a particular analysis?

Author's reply:

The present version includes Table 6, which summarizes results for various pairs of variables.
